# Cell Densities in the Mouse Brain: A Systematic Review

**DOI:** 10.3389/fnana.2018.00083

**Published:** 2018-10-23

**Authors:** Daniel Keller, Csaba Erö, Henry Markram

**Affiliations:** Blue Brain Project, École Polytechnique Fédérale de Lausanne, Geneva, Switzerland

**Keywords:** cell density, mouse brain, brain regions, stereology, whole-brain atlas

## Abstract

The mouse brain is the most extensively studied brain of all species. We performed an exhaustive review of the literature to establish our current state of knowledge on cell numbers in mouse brain regions, arguably the most fundamental property to measure when attempting to understand a brain. The synthesized information, collected in one place, can be used by both theorists and experimentalists. Although for commonly-studied regions cell densities could be obtained for principal cell types, overall we know very little about how many cells are present in most brain regions and even less about cell-type specific densities. There is also substantial variation in cell density values obtained from different sources. This suggests that we need a new approach to obtain cell density datasets for the mouse brain.

## Introduction

Here we review what is actually known about total cell density and cell-type specific density in the whole mouse brain and in the different brain regions. To our knowledge, this is the first attempt to determine the full corpus of knowledge available today on cell densities for all regions of the mouse brain.

Establishing the numbers of cells in the brain is arguably the first step when developing a complete catalog of cell-types as proposed by the Cell Census Network effort of the BRAIN Initiative (Bargmann et al., 2014). Cell density data could provide constraints when attempting to understand the brain's organization and function. For example, they could be used to help quantify the differences between species, calculate the energy consumption in different regions, understand the role of specific types of neurons in different brain regions and the computational properties and capabilities of different brain regions, and map and understand the connectome (Srinivasan et al., 2015; Weigand et al., 2017). They are also essential in integrative attempts to reconstruct and simulate the brain (Markram, 2006; Markram et al., 2015).

Today, there are some estimates for the cell numbers in the whole brain of several species (Lange, 1975; Herculano-Houzel et al., 2006, 2007), but how much do we know about the density of cells in each brain region? How much do we know about the density of specific cell-types in each brain region? How reliable are the estimates that have been reported? To answer these questions, we drew from the corpus of experimental literature that either provided such data directly or measurements that could be readily transformed into cell density. We adopted a standardized approach for transforming and analyzing data, and quantified the variability for different estimates of neuron density within regions and between different regions.

Cells within the central nervous system can be broadly classified into neurons, glia, endothelial cells, and pericytes (Kandel, 2013). These categories in turn are subject to further sub-classification. Neurons are functionally categorized according to whether they are excitatory, inhibitory, or neuromodulatory, electrically categorized by the manner in which they respond to stimulation, and anatomically categorized by the morphology of the dendrites and axons, their afferent and efferent projections and the specific proteins and genes that they express. Inhibitory interneurons are functionally categorized by their hypothesized impact on excitatory neurons, electrically categorized by their response properties, and anatomically categorized by their morphology and by their expression of specific proteins and genes (Markram et al., 2004). Neuromodulatory neurons are categorized according to the neuromodulatory neurotransmitter they produce (dopamine, noradrenaline, serotonin, histamine, etc…) and the brain regions where they reside in. Glia can be divided into two principal types: microglia and macroglia. Microglia play an immunological and scavenging role in the brain. Macroglia on the other hand can play diverse roles. Astrocytes, for example, support the neurotransmitter, ionic and metabolic needs of neurons; oligodendrocytes support the axons by producing myelin; endothelial cells line larger blood vessels, and pericytes support the smaller capillaries. Cells of different types can often be distinguished by marker expression and morphology.

We performed a large-scale search for reports cell densities and numbers within the adult mouse brain, focusing on what is known about the densities of neurons, endothelial cells, astrocytes, oligodendrocytes, and microglia. In principle, astrocytes, oligodendrocytes, microglia, and the other glia subtypes should sum up to equal the total glial densities reported in the literature. When possible, neurons were divided into excitatory cells and inhibitory interneurons. Numbers for other region-specific neuron types were also examined: dopaminergic neurons in the basal ganglia, granule cells in the hippocampus, and Purkinje and granule cells in the cerebellum.

Within the literature, cell density is estimated using several methodologies, including stereology, optical fractionation, simple estimation from micrographs, and whole-brain imaging. As discussed in the methods section, each approach has different degrees of reliability. This review combines and compares numbers obtained by disparate methods.

## Methods

We strove to consider all published literature and dissertations which reported cell density and numbers for particular structures, which amounts to a corpus of literature spanning 60 years up to mid-2018. Papers were first identified using Pubmed and Google Scholar. References within identified papers were traced to identify additional sources. In some reports, it was necessary to extract data from bar plots by digitizing the graph and measuring the image.

The mouse strain, age, and gender were chosen in order to constrain the scope of the review. Cell densities can vary somewhat among mice of different strains (Nelson et al., 1998; Mozhui et al., 2007). We chose C57Bl/6 as the reference strain, as it is most frequently employed in experiments, though for some parameters only data from other strains was available. Male adult animals aged 55 days were preferentially studied, though sometimes only data from other ages or females was available. In such instances, data from animals older than P28 was preferentially used, since before this age the animals are still developing and rapidly changing.

Different markers which identify specific cell populations are generally used in studies that report cell density numbers. Most commonly, NeuN is used to mark neurons, S100β, CNPase, and Iba1 to mark oligodendrocytes, and S100β, Glial Fibrillary Acidic Protein (GFAP) and glutamine synthetase (GS) to mark astrocytes. However, some markers exhibit either incomplete coverage of the target cell population or label multiple cell types. For example, not all astrocytes express GFAP in the cortex and hippocampus of adult animals (Ling and Leblond, 1973; Kitamura et al., 1987; Walz and Lang, 1998; Savchenko et al., 2000). Therefore, relying only on this marker to identify astrocytes results in systematic undercounting (Wu et al., 2005) In addition to oligodendrocytes, antibodies directed against S100β, a member of the large S100 calcium-binding protein family, also label astrocytes (Grosche et al., 2013). In fact, S100β is expressed in more astrocytes than is GFAP (Rickmann and Wolff, 1995a,b). Even using light microscopy, oligodendrocytes and astrocytes can be distinguished by morphology, allowing S100β to be used to obtain density estimates for both types of cells. Oligodendrocytes also express CNPase (2′,3′-Cyclic-nucleotide 3′-phosphodiesterase), and the promoter element is commonly used to drive GFP expression for identifying oligodendrocyte populations (Vinet et al., 2010). Activated microglia are commonly labeled with Iba1 (Jinno et al., 2007).

Relevant papers reported several types of numbers: cell density, numbers of neurons in a particular structure as counted stereologically, or numbers of neurons in a slice or sampling region. Most studies do not directly study adult wild type mice as an end in itself, but instead test an experimental modification. In such cases, we could still use the data from the control cases corresponding to the wild type condition. When multiple measurements from mice were available from a single source, the mean value of all measurements was used. We should emphasize that many of the density numbers reported here were not directly reported in the original publications but rather are our own estimates derived from the data in the original paper. Calculations are provided in the Supplemental Material.

The most accurate and highly-refined method of measuring cell densities is via stereology, which uses statistical extrapolation during cell counting to obtain an estimate (Mouton, 2014). Stereologists count cells in a measured region directly, and generally have access to relevant parameters such as the degree of tissue shrinkage.

Sometimes cell density estimates are not given explicitly, but rather reported in two-dimensional (2D) micrographs of a single slice without any further correction (see Supplementary Material). It was thus necessary to divide by slice thickness to obtain volumetric density. This was done to analyze some measurements (Trune, 1982; Shimada et al., 1992; Caggiano and Brunjes, 1993; Anderson et al., 1998; Geisert et al., 2002; Angenstein et al., 2008; Lorke et al., 2008; Pernet et al., 2008; Jahanshahi et al., 2009; Pott et al., 2009; Richard et al., 2010; Binder et al., 2011; Sargeant et al., 2011 Huang et al., 2012; Barrera et al., 2013; Pitts et al., 2013; Reyes-Haro et al., 2013; Schmid et al., 2013; Chen et al., 2014). The corresponding estimates can be expected to be influenced by error in the slice thickness as well as by occlusion effects of overlapping cells. Furthermore, if cut neuronal cell bodies at the edges are not explicitly compensated for, the neuronal density estimate can be inaccurate. Edge effect error can be expected to decrease with increasing slice thickness.

In certain cases, only an absolute cell number for a given structure was reported (Heumann and Rabinowicz, 1980; Sturrock, 1989, 1991; Bayer et al., 1994; Bing et al., 1994; Scott et al., 1994; Jeffrey et al., 1995; Hadj-Sahraoui et al., 1996; Kempermann et al., 1997; Cunningham et al., 1999; Doulazmi et al., 1999; Andsberg et al., 2001; Geisert et al., 2002; Wirenfeldt et al., 2003; Woodruff-Pak, 2006; Zhang et al., 2007, 2012; Fabricius et al., 2008; Morris et al., 2008; Burguet et al., 2009; Dursun et al., 2011; Kuronen et al., 2012; Mouton, 2014). For example, the isotropic fractionator is a method that produces cell counts by first transforming dissected brain regions into isotropic suspensions of brain nuclei, which can be then counted and classified as neuronal or non-neuronal (Herculano-Houzel and Lent, 2005). Total cell counts for structures are reported. To obtain cell density when only an absolute number in a structure is given, it is necessary to divide the number in the structure by the volume of the structure. Volume estimates were taken from either the source paper, when possible, or from other sources such as the Allen Brain Atlas (Lein et al., 2007). When the volume comes from a different source, it is potentially influenced by subjective differences in identifying the boundaries of the structures.

Some experimental approaches may themselves be subject to inherent bias. For example, if no shrinkage factor is given when counting cells in a block of fixed tissue, the resulting cell densities most likely are overestimations. Using markers that do not stain every cell in a target population will produce systematic underestimation of the target population. It is difficult to compensate for such bias in a rigorous manner, given that the parameters needed to compensate are largely unknown. Systematic bias therefore likely contributes to the variability observed when data obtained using different approaches is combined.

Papers were screened according to whether the results were in a plausible range of values. If at least three papers reported a mean value for a particular parameter in a given region and a candidate paper reported a value more than a factor of two different than the consensus, the candidate paper was omitted for further consideration.

When combining the results of different studies, we assign equal weight per source. It is true that the number of samples used to estimate a value vary from source to source, but the methods used were so different as to preclude finer-grained pooling. We do not believe that the policy of equal weight per source introduces systematic bias into the review procedure.

The principal way of evaluating the results is by assessing the mean and standard deviation of estimates within individual brain regions and the standard deviation between average values in different brain regions. We also evaluate the degree of coverage of principal cell types for which data could be found in the major brain regions.

## Results

We screened 101 papers reporting density-related information in the mouse. Of those papers, four were culled according to the removal criteria outlined above. Neuron densities are best characterized in the cortex, hippocampus, cerebellum, and striatum. Data for other subcortical structures and nuclei are sparse.

### Neocortex

The cortex is located on the outside of the brain and is made of gray matter containing many neuronal cell bodies and relatively few myelinated axons. It is composed of multiple layers, each with different densities. Most excitatory cells are pyramidal cells, and non-neuronal cells outnumber neurons. Inhibitory GABAergic interneurons make up 15–20% of all cortical neurons (Gentet et al., 2000).

The density of neurons also varies across cortical areas. Table 1 lists relevant densities for cortex. Estimates of neuronal density range from 48,000 cells/mm^3^ in orbital cortex to 155,000 cells/mm^3^in visual cortex (see Table 1). Averaging neurons across all areas in Table 1 gives an average of 92,616 ± 25,000 cells/mm^3^ (mean ± std). In Table 1, estimates for total cell density are generally less than the sum of neuronal and non-neuronal densities, but larger than neuronal densities found in other work. The total cell density estimates were obtained using propidium iodide to stain all cellular nuclei in the CUBIC-X protocol (Murakami et al., 2018). The estimates used in the table may be better regarded as intermediate between total neurons and total cells, or a lower bound on the number of total cells.

**Table 1:** Estimates of Cortical Density.

**Region**	**Neuron density**	**Non-neuronal cell density**	**Total cell density**	**Interneuron subtypes**	**Astrocytes**	**Oligodendrocytes**	**Microglia**
Cortex (General)	92,000 (Schüz and Palm, 1989)	43,000 (Tsai et al., 2009)	127,870 (Murakami et al., 2018)	2,903 PV, 4,877 SST, 1,935 VIP (Kim et al., 2017)	15,696 S-100β (Grosche et al., 2013)	12,500 (Rockland and DeFelipe, 2012)	6,500 (Nimmerjahn et al., 2005) 6,500 (Lawson et al., 1990)
Frontal cortex	66,771^*^ 104,000 (Rajkowska et al., 2016) 123,000 (Schmid et al., 2013) 190,000 (Rockel et al., 1980) **Mean and STD: 121,000** ± **52,000**	79,393^*^	104,100 (Murakami et al., 2018)	863 PV, 1,749 SST, 2,598 VIP (Kim et al., 2017)	7,000 GFAP+ (Xu et al., 2007) 52,000 GFAP+ (Rajkowska et al., 2016) **Mean and STD: 29,500 ± 31,820**	87,803 (Duque et al., 2012)	6,200 (Lawson et al., 1990)
Posterior parietal association areas	76,588^*^ 230,000 (Rockel et al., 1980) **Mean and STD:** **150,000** ± **100,000**	115,318^*^	150,530 (Murakami et al., 2018)	4,452 PV, 5,140 SST, 2,512 VIP (Kim et al., 2017)	–	—	6,124 (San Jose et al., 2001) 6,900 (Lawson et al., 1990) **Mean and STD:** **6,512** ± **549**
Visual areas	155,426^*^ 214,000 (Rockel et al., 1980) 92,400 (Cragg, 1967) 194,000 (Heumann et al., 1977) **Mean and STD:** **164,000** ± **50000**	169,804^*^	145,170 (Murakami et al., 2018)	4,886 PV, 5,028 SST, 2,964 VIP (Kim et al., 2017)	49,600 S-100β (Argandoña et al., 2009)	10,000 (Tremblay et al., 2012)	7,250 (Tremblay et al., 2012)
Anterior cingulate area	76,747^*^	127,034^*^	133,600 (Murakami et al., 2018)	3,882 PV, 4,399 SST, 2,597 VIP (Kim et al., 2017)	—	—	5,600 (Lawson et al., 1990)
Primary motor area	74,775^*^	90,172^*^	117,480 (Murakami et al., 2018)	4,060 PV, 4,241 SST, 2,088 VIP (Kim et al., 2017)	—	—	—
–(Motor)	124,000 (Schmid et al., 2013) 146,000 (Rockel et al., 1980) **Mean and STD:** **135,000** ± **16,000**	—	—	13,415 GAD+ (Irintchev et al., 2005) 6,707 PV (Irintchev et al., 2005) 1,500 PV (Schmid et al., 2013) 3,366 CB (Irintchev et al., 2005)	15,000 S-100β (Schmid et al., 2013)	15,000 (Irintchev et al., 2005) 64,274 (Duque et al., 2012) **Mean and STD: 39,637** ± **34,842**	14,700 (Irintchev et al., 2005)
Secondary Motor Area	135,801^*^	171,184^*^	—	3,763 PV, 3,905 SST, 2,212 VIP (Kim et al., 2017)	—	—	—
Sensorimotor Cortex	127,000 Sensory (Schmid et al., 2013)	—	123,970 (Murakami et al., 2018)	207 nNOS (Chen et al., 2014) 2,200 PV (Sensory) (Schmid et al., 2013) 3,932 PV, 4,094 SST, 2,142 VIP (Kim et al., 2017)	11,000 Sensory S-100β (Schmid et al., 2013)	—	16,667 (Chen et al., 2014) 2254 (Lorke et al., 2008) **Mean and STD:** **9,461** ± **10,192**
Somatosensory	111,319^*^ 110,000 (Irintchev et al., 2005) 110,000 (Rockel et al., 1980) **Mean and STD:** **110,000** ± **1,000**	126,513^*^	127,850 (Murakami et al., 2018)	13,659 GAD (Irintchev et al., 2005) 6,951 PV (Irintchev et al., 2005) 4580 PV (Ransome and Turnley, 2005) 4,390 CB (Irintchev et al., 2005) 9560 CB (Ransome and Turnley, 2005) 5,490 PV, 4,586 SST, 1,987 VIP (Kim et al., 2017) 3840 SST+ (Ransome and Turnley, 2005) 2230 CR (Ransome and Turnley, 2005)	9,548 SR-101 superficial layers (Hill and Grutzendler, 2014) 18400 GFAP L1, 25000 GFAP L6b (Ransome and Turnley, 2005)	15,000 (Irintchev et al., 2005) 2,589 in superficial layers (Hill and Grutzendler, 2014) **Mean and STD: 8,795** ± **8,776**	6,185 (Irintchev et al., 2005) 2,422 (Lorke et al., 2008) **Mean and STD:** **4,304** ± **2,661**
Somatosensory, Barrel	87,115^*^	100,834^*^	126,710 (Murakami et al., 2018)	5,894 PV, 4,450 SST, 1,877 VIP (Kim et al., 2017)	—	29,674 (Barrera et al., 2013)	—
Supplemental somatosensory area	82,433^*^	103,812^*^	128,470 (Murakami et al., 2018)	4,837 PV, 4,705 SST, 1,934 VIP (Kim et al., 2017)	—	—	—
Auditory areas	109,730^*^	115,847^*^	128,400 (Murakami et al., 2018)	4,345 PV, 4,793 SST, 2,142 VIP (Kim et al., 2017)	—	4,000 (Tremblay et al., 2012)	7,500 (Tremblay et al., 2012)
Infralimbic area	85,755^*^	133,987^*^	116,990 (Murakami et al., 2018)	1,926 PV, 7,942 SST, 1,669 VIP (Kim et al., 2017)	—	—	—
Orbital area	48,109^*^	80,048^*^	139,950 (Murakami et al., 2018)	3,900 PV, 4,892 SST, 1,859 VIP (Kim et al., 2017)	—	—	—
Agranular insular area	75,506^*^	104,423^*^	109,470 (Murakami et al., 2018)	2,032 PV, 4,626 SST, 1,580 VIP (Kim et al., 2017)	—	—	—
Retrosplenial area	98,148^*^	—	188,810 (Murakami et al., 2018)	5,276 PV, 5,044 SST, 2,770 VIP (Kim et al., 2017)	—	—	—
Ectorhinal area	69,066^*^	—	109,100 (Murakami et al., 2018)	1,715 PV, 5,296 SST, 2,973 VIP (Kim et al., 2017)	—	—	—
Piriform area	74,253^*^	—	101,260 (Murakami et al., 2018)	669 PV, 4,376 SST, 895 VIP (Kim et al., 2017)	—	—	8,100 (Lawson et al., 1990)

The visual cortex has the highest neuron density. Astrocyte density is highest in visual cortex, while oligodendrocyte density is highest in frontal cortex. In the rest of the brain, microglia, and oligodendrocyte densities fall into a characteristic range without much variation between brain structures.

* *Densities from (Herculano-Houzel et al., 2013). The marker used to identify inhibitory interneuron subtypes is Glutamate Decarboxylase (GAD). Additional neuron markers mentioned are: Calbindin (CB), parvalbumin (PV), and neuronal nitric oxide synthase (nNOS)*.

A variety of non-neuronal cells are present in cortex. Averaging the obtained astrocytic densities across available reports listed in Table 1 yields 20,000 ± 13,000 cells/mm^3^ (mean ± std); a mean ratio of astrocytes to neurons of 0.2 ± 0.1. Overall in cortex, endothelial cell density is 70,000 cells/mm^3^ (Niedowicz et al., 2014) and total glia density is 36,364 cells/mm^3^ (Geisert et al., 2002). Estimates of oligodendrocyte density varied widely, even for the same region. The average cortical density of microglia in Table 1 is 8,500 ± 3,900 cells/mm^3^. In the lesser-studied cortical regions, data for astrocytes, oligodendrocytes, and microglia is not readily available.

**Figure 1:**
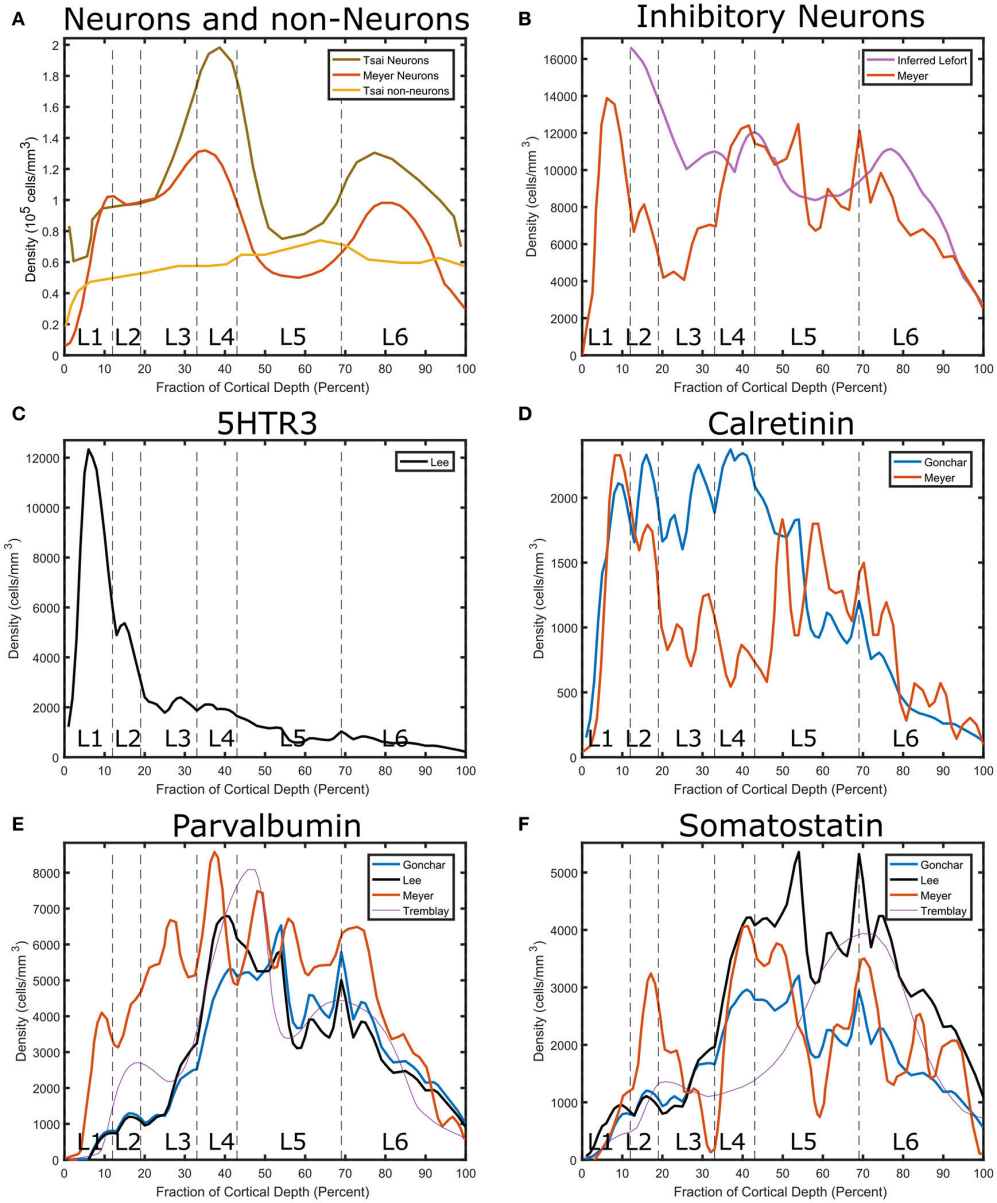
Layer-specific estimates of cell densities in granular cortex (Tsai et al., 2009) barrel cortex S1 (Meyer et al., 2010) and visual cortex (Gonchar et al., 2007) and somatosensory cortex (Lee et al., 2010,?). **(A)** Comparison between neurons and other types. Both data sets show a peak in layer 4 neuronal density and have a secondary peak in layer 6. **(B)** Interneuron Density. The layer-specific fraction of interneurons reported in Lefort et al. (2009) was multiplied by the neuron density reported in Tsai et al. (2009) for comparison to the Meyer data. Interneurons exhibit a peak in Layer 1, which is however not included in the Lefort data set. To obtain marker specific interneuron densities, the fraction of interneurons in each layer reported in Lee et al. (2010) and Gonchar et al. (2007) was multiplied by the interneuron density reported by Meyer. **(C)** Serotonin receptor distribution. It exhibits a peak in the superficial layers. The normalized Tremblay inhibitory density sum was scaled to match the inhibitory density from Meyer. **(D)** Calretinin distribution. It shows a peak in the central part of the cortex. **(E)** Parvalbumin distribution. **(F)** Somatostatin distribution.

Neuronal densities vary across layers (Figure 1A). Excitatory neuron density peaks in the upper part of layer 4 and has a secondary peak in the deeper layers, while inhibitory neuron density is highest in layer 2 (Figure 1B). The excitatory peak approached 200,000 cells/mm^3^ while the layer 2 inhibitory peak was more than 10,000 cells/mm^3^ (Meyer et al., 2010, 2011).

Cortical interneurons can be coarsely categorized into three subtypes depending on primary markers expressed: the neuropeptide somatostatin (SST), parvalbumin (PV), and the ionotropic serotonin receptor 5HT3a (5HT3aR) (Rudy et al., 2011). The SST subtype comprises 30% of GABAergic neurons and the PV type is about 40% (ibid.). The 5HT3aR group forms the remaining 30% of the total interneuronal population. The 5HT3aR group is quite varied and is equally divided into all of the neurons that express the vasointestinal peptide (VIP) and another subgroup that does not express VIP (e.g., neurogliaform cells) (Rudy et al., 2011).

Layer-specific percentage breakdowns in interneuron types are obtained from the immunohistochemistry studies (Lee et al., 2010). We combine this information with an estimate of absolute neuron number to estimate the densities of interneuron types in different layers of cortex (Figures 1C–F). Neurons expressing 5HTR3A peak in layer 1. Neurons expressing calretinin (CR) exhibit different profiles depending on the source. The consensus from multiple sources is that PV neuron density peaks in layer 4 and 5a, while SST-expressing interneurons peak in the deep layers. While cholinergic interneurons do exist in the cortex, their density is much lower than the ones in these main categories.

Cells expressing 5HTR3A, SST, PV, and calbindin (CB) map to several cell sub-types (Table 2), with every subtype having its own morphological and electrical properties. Each marker in itself is not sufficient to distinguish the sub-class of interneuron.

**Table 2:** Mapping of marker-expressing interneurons to standard cell types in cortex.

**Marker expressed**	**Percent of interneurons**	**Morphology**	**Electrophysiology**
5HTR3A Serotonin Receptor	5	Heterogenous: bipolar/double bouquet /bitufted/neurogliaform	BS/ high rheobase, delayed firing, marked frequency adaptation
Calretinin	15	Small Bipolar	RS/BS
Parvalbumin	50	Large/ Nest Basket Chandelier	FS/RS FS
Somatostatin	30	Small Basket Martinotti	RS BS
Cholecystokinin	13	Basket	RS, Accommodating

*BS, Burst spiking; RS, Regular Spiking; FS, Fast Spiking. The table is based on (Cauli et al., 1997; Wonders and Anderson, 2006; Vucurovic et al., 2010; Rudy et al., 2011; Bartos and Elgueta, 2012). Cholecystokinin (CCK) percentages were obtained by multiplying the ratio of CCK to PV in somatosensory cortex (Whissell et al., 2015) by the parvalbumin percentage. Note that not all categories are mutually exclusive*.

If more genes are used, more subtypes can be distinguished. Transcriptome-based studies have identified as many as 16 different types of interneurons in the cortex (Zeisel et al., 2015), which implies that further sub-division beyond common inhibitory markers is possible.

### Hippocampus and associated areas

The hippocampus is part of the limbic system and plays a role in memory consolidation and spatial navigation. The majority of its input comes from the closely-associated entorhinal cortex. Neuron densities in the main part of hippocampus are lower than in cortex. Reported estimate of neuron density varied widely, as Table 3 illustrates. Densities for most cell types were available in the hippocampus proper, though in the subiculum and entorhinal cortex glia density information was sparse.

**Table 3:** Cell densities in hippocampus and associated regions.

**Region**	**Neuron density**	**Interneuron subtypes**	**Astrocytes**	**Microglia**
Overall	20,848 (Geisert et al., 2002)	3,100 (Schmalbach et al., 2015) 2,163 GAD67+ (Neddens and Buonanno, 2010) **Mean and STD: 2,600** ± **600** 1,219 PV+ (Neddens and Buonanno, 2010) 1,260 SST+ (Neddens and Buonanno, 2010) 201 CCK+ (Neddens and Buonanno, 2010) 1,274 nNOS+ (Neddens and Buonanno, 2010) 8,200 Reelin+ (Schmalbach et al., 2015) 1,480 PV,4,402 SST, 1,060 VIP (Kim et al., 2017)	29,008 GFAP+ (Shimada et al., 1992) 4,811 GFAP+ (Geisert et al., 2002) 12,226 S-100β (Schmalbach et al., 2015) 20,904 S-100β (Grosche et al., 2013) 4,353 S-100β (Geisert et al., 2002) **Mean and STD: 14,260** ± **1,060**	4,353 (Geisert et al., 2002) 2,143 (Schmalbach et al., 2015) **Mean and STD: 3,248** ± **1,563**
CA1	65,000 (Hlatky et al., 2003)	1,480 PV+ (Pitts et al., 2013) 1,158 PV+ (Neddens and Buonanno, 2010) 1,182 SST+ (Neddens and Buonanno, 2010) 273 (Neddens and Buonanno, 2010) 927 nNOS+ (Neddens and Buonanno, 2010) 2,319 GAD67+ (Neddens and Buonanno, 2010) 898 PV, 2,458 SST, 821 VIP (Kim et al., 2017)	43,950 GFAP+ (Wu et al., 2005) 62,590 GS+ (Wu et al., 2005) 9,570 GS+ (Olabarria et al., 2011) 25,300 S100β (Ogata and Kosaka, 2002) 64,650 S100β (Wu et al., 2005) **Mean and STD: 41,200** ± **23,800**	22,300 18800 Mac1+ (Long et al., 1998)
Dorsal CA1	447,500 (pyramidal neurons) (Jinno and Kosaka, 2010) 165,742 (Miranda et al., 2009) **Mean and STD:** **307,000** ± **200,000**	–	23,700 S100β (Ogata and Kosaka, 2002)	8,500 (Lawson et al., 1990) 6,120 (Jinno et al., 2007) **Mean and STD: 7,310** ± **1,683**
Ventral Ca1	180,500 (pyramidal neurons) (Jinno and Kosaka, 2010)	–	26,900 S100β (Ogata and Kosaka, 2002)	5,940 (Jinno et al., 2007)
CA2	—	1,342 PV, 1,760 SST, 546 VIP (Kim et al., 2017)	39,150 GFAP+ 61,420 GS+ 63,510 S100β (Wu et al., 2005) **Mean and STD: 54,690** ± **13,500**	–
CA3	123,280 (Fabricius et al., 2008) 168,800 (pyramidal neurons) (Jinno and Kosaka, 2010) 154,740 (Kurt et al., 2004) 110,000 (Hlatky et al., 2003) **Mean and STD:** **140,000** ± **27,000**	2171 PV+ (Pitts et al., 2013) 1127 PV+ (Neddens and Buonanno, 2010) 1162 SST+ (Neddens and Buonanno, 2010) 217 CCK+ (Neddens and Buonanno, 2010) 1587 nNOS+ (Neddens and Buonanno, 2010) 2130 GAD67+ (Neddens and Buonanno, 2010) 1,098 PV, 3,815 SST, 367 VIP (Kim et al., 2017)	24,400 S100β (Ogata and Kosaka, 2002) 34350 GFAP+ 54390 GS+ 58450 S100β (Wu et al., 2005) **Mean and STD: 42,900** ± **16,200**	5,350 (Jinno et al., 2007) 2,310 (Lorke et al., 2008) **Mean and STD: 3,830** ± **2,150**
Dorsal CA3	—	—	26,400 S100β (Ogata and Kosaka, 2002)	5,990 (Jinno et al., 2007)
Ventral CA3	—	—	22,400 S100β (Ogata and Kosaka, 2002)	4,760 (Jinno et al., 2007)
CA4	37,200 (Fabricius et al., 2008)	–	—	32,200 (Long et al., 1998)
Dentate Gyrus	In stratum granulosum: 625,080 (Kurt et al., 2004) 484,890 (Fabricius et al., 2008) 393,750 (Rajkowska et al., 2016) **Mean and STD:** **501,240** ± **116,530** 11,980 (granule cells) (Jenrow et al., 2006) 9,087 (Kempermann, Kuhn, and Gage 1997) 2,737 (Granule cells only) (Farrar et al., 2005) **Mean and STD of Granule Cells: 7,935** ± **4,728**	493 PV+ (Pitts et al., 2013) 745 PV+ (Neddens and Buonanno, 2010) 400 PV+ (Fasulo et al., 2017) 1,050 SST+ (Neddens and Buonanno, 2010) 53 CCK+ (Neddens and Buonanno, 2010) 887 nNOS+ (Neddens and Buonanno, 2010) 1,498 GAD67+ (Neddens and Buonanno, 2010) 274 PV, 1,781 SST, 362 VIP (Kim et al., 2017)	36,000 GFAP+ (Rajkowska et al., 2016) 24,300 S100β (Ogata and Kosaka, 2002) 5,533 GS+ (Olabarria et al., 2011) **Mean and STD: 22,000** ± **15,000**	18,800 Mac1+ (Long et al., 1998) 3,968 (Wirenfeldt et al., 2003) 12,000 (Lawson et al., 1990) **Mean and STD:** **11,589** ± **7,425**
Dorsal Dentate Gyrus	—	—	19,900 S100β (Ogata and Kosaka, 2002)	5,560 (Jinno et al., 2007)
Ventral Dentate Gyrus	—	—	28,700 S100β (Ogata and Kosaka, 2002)	5,600 (Jinno et al., 2007)
Subiculum	128,526 (Trujillo-Estrada et al., 2014) 72,110 (Fabricius et al., 2008) **Mean and STD:** **100,318** ± **39,892**	2,048 PV+ (Neddens and Buonanno, 2010) 16,991 PV+ (Trujillo-Estrada et al., 2014) 266 CCK+ (Neddens and Buonanno, 2010) 1,753 SST+ (Neddens and Buonanno, 2010) 1,611 nNOS+ (Neddens and Buonanno, 2010) 2,806 GAD67+ (Neddens and Buonanno, 2010) 2,790 PV, 6,407 SST, 1,314 VIP (Kim et al., 2017)	—	–
Entorhinal	100,130 (Herculano-Houzel et al., 2013) 100,000 pyramidal layers I-III (Moreno-Gonzalez et al., 2009) 80,000 pyramidal layers IV-VI (Moreno-Gonzalez et al., 2009)	13,000 SST+ (Moreno-Gonzalez et al., 2009) 3,700 NPY+ (Moreno-Gonzalez et al., 2009) 4,300 PV+ (Moreno-Gonzalez et al., 2009) 1,319 PV, 6,406 SST, 1,392 VIP (Kim et al., 2017)	—	—

*Reported neuron densities vary markedly, though this might be attributable to differences in the counting region volume and method as explained in the text. Astrocyte and microglia densities have median values of 24,400 and 5,990 cells/mm^3^, respectively. CCK, Cholecystokinin; NPY, neuropeptide Y; nNOS, neuronal nitric oxide synthase. Mean and standard deviation were only provided when multiple numbers were available for cells of the same type*.

The two major components of the hippocampus are the cornus ammonus (CA) and the dentate gyrus. The hippocampus shape has been compared to a ram's horn, from which the name cornus ammonus derives. The CA abbreviation is used to name the hippocampus subfields CA1-4. There are different strata within these structures, the stratum oriens, pyramidale, lucidum, radiatum, lacunosum, and moleculare. Cell bodies are not distributed uniformly, but rather the composition varies according to the strata. Few experimentalists make this distinction when determining cell densities, and cell densities therefore refer to the coarser regional designations.

Astrocyte estimates obtained using different markers were averaged together to obtain mean estimates of astrocytes in each region. This is likely to underestimate true density, since not all markers are expressed in all astrocytes. Still, the overall astrocyte to neuron ratio, 0.68, is higher than in cortex.

Oligodendrocytes in the hippocampus fall into at least four different types, each distributed differently according to strata (Vinet et al., 2010). Each has characteristic distributions and density. However, absolute numerical densities of oligodendrocytes in the hippocampus are hard to come by. Therefore, they are not included in Table 3.

### Cerebellum

The cerebellum, present in the hindbrain, plays an important role in motor control. It has a high density of neurons. Indeed, it represents half of the total neuron number in the brain. There are two main types of neurons in the cerebellum: granule cells and Purkinje Cells. While granule cells are small, Purkinje Cells are large and only found in a single thin layer. Due to the ambiguity inherent in measuring the volume of the Purkinje Cell layer we took the Purkinje Cell density to be number with respect to the area of the Purkinje Cell layer interface rather than to a volume. The units were accordingly neurons/ mm^2^. Aside from this issue, the cerebellum is relatively simple. There are fewer primary cell types in the cerebellum, and the circuit layout follows a stereotyped pattern.

Granule cell estimates vary markedly, possibly due to the difficulty in counting them. Their small size and high density mean that cells may become difficult to distinguish. Astrocyte and oligodendrocyte density information was generally sparse for the cerebellum. The ratio of astrocytes to neurons was lower in cerebellum than in cortex or hippocampus.

### Subcortical structures

Subcortical structures are quite heterogeneous. Included in this category are various nuclei, the striatum, the thalamus, and the hypothalamus (Table 5). Although some such as the olfactory bulb and nucleus, amygdala, and hypothalamus have neuronal densities higher than cortex, others such as the striatum, substantia nigra, and superior colliculus have the same or lower density.

**Table 4:** Cell densities in cerebellum and associated structures.

**Region**	**Neuron density**	**Astrocytes**	**Oligodendrocytes**	**Microglia**
Overall	1,210 Purkinje Cells/mm^2^ (Zanjani et al., 1997) 1,100 Purkinje Cells/mm^2^ (Woodruff-Pak, 2006) 1,050 Purkinje Cells/mm^2^ (Doulazmi et al., 1999) 846 Purkinje Cells/mm^2^ (Hadj-Sahraoui et al., 1996) 1,230 Purkinje Cells/mm^2^ (Isaacs and Abbott, 1995) 1,263 Purkinje Cells/mm^2^ Purkinje (Fan et al., 2001) **Mean and STD: 1,120** ± **160 PCs/mm**^2^ Volumetric estimates: 25,570 Purkinje Cells/mm^3^ (Lange, 1975) 17,000 Purkinje Cells/mm^3^ (Förster, 2008) 14,000 Stellate and Basket Cells/mm^3^ (Förster, 2008)	1,512 (Rockland and DeFelipe, 2012)	15,000 (Förster, 2008) 12,500 (San Jose et al., 2001) 1840.8 CC+ (Relucio, 2011) **Mean and STD Regular Oligodendrocytes:** **13,750** ± **1,768**	9,090 (Rockland and DeFelipe, 2012) 8,158 (Journiac et al., 2005) **Mean and STD: 8,624** ± **659**
Granular layer	6,562,500 granule cells (Zanjani et al., 1997) 500,000 (Steen, 2006) **Mean and STD: 4,000,000** ± **4,000,000**	9,000 S100 (Steen, 2006) 5,400 S100 (Förster, 2008) **Mean and STD: 7,200** ± **2,546**	6,000 (Steen, 2006) 5,400 (Förster, 2008) **Mean and STD: 5,700** ± **424**	7,000 (Steen, 2006) 3,500 (Förster, 2008) 3,281 (Vela et al., 1995) 3,200 (Lawson et al., 1990) 1,116 (Lorke et al., 2008) **Mean and STD: 3,619** ± **2,121**
Molecular layer	120,110 (Lange, 1975)	—	—	3,500 (Förster, 2008) 2,200 (Lawson et al., 1990) 1,367 (Vela et al., 1995) **Mean and STD: 2,356** ± **1,075**
Cerebellar nuclei	—	—	—	7,300 (Lawson et al., 1990)
Vestibular nuclei	2,976 Lateral Vestibular Nucleus (Sturrock, 1989)	—	—	5,632 (Vela et al., 1995)
Inferior Olive	24,598 (Zanjani et al., 1997)	—	—	—

*Purkinje Cell density was converted to cells/mm^2^ except in cases where the original paper only reported a volumetric density*.

**Table 5:** Cell densities for subcortical structures.

**Region**	**Neuron density**	Total cells^*^	**Interneuron subtypes**	**Total glia estimate (Cells/mm^3^)**	**Astrocytes**	**Oligodendrocytes**	**Microglia**
Main olfactory bulb	150,000 (Quay and Wilhoft, 1964) 203,000 (Parrish-Aungst et al., 2007) **Mean and STD:** **177,000** ± **37,000** GL 410,000 EPL 42,000 MCL 270,000 IPL 150,000 GCL 250,000 (Parrish-Aungst et al., 2007) 404,000 GCL (Richard et al., 2010) 53,850 NeuN+PG+ in GCL (Richard et al., 2010) 15,900 TH+ PG+ in GCL (Richard et al., 2010)	ONL 130,000 GL 630,000 EPL 70,000 MCL 360,000 IPL 150,000 GCL 410,000 SEL 8,300,000 430,000 (Parrish-Aungst et al., 2007) 176,964 (Murakami et al., 2018)	584 PV, 6,235 SST, 3,796 VIP (Kim et al., 2017)	—	—	—	11,500 (Lawson et al., 1990) SBE 7,300 Ramified, 5,266 Ameboid GCL 6,666 Ramified, 500 Ameboid MCL 7,666 Ramified EPL 9,333 Ramified, 500 Ameboid GLM 6,666 Ramified (Caggiano and Brunjes, 1993)
Olfactory Nucleus	189,900 (Brunjes et al., 2011)	—	630 PV, 3,818 SST, 891 VIP (Kim et al., 2017)	—	—	—	6,700 (Lawson et al., 1990)
Amygdala MePD	—	149,850 (Murakami et al., 2018)	1,238 PV, 13,036 SST, 3,095 VIP (Kim et al., 2017)	—	38,200 GFAP+ (male) (Johnson et al., 2012)	—	—
Amygdala (basolateral)	194,000 (Mozhui et al., 2007)	104,870 (Murakami et al., 2018)	1,177 PV, 5,951 SST, 1,930 VIP (Kim et al., 2017) 1,400 PV+ (Fasulo et al., 2017) 3,920 nNOS (Wang et al., 2017)	28,000 (Mozhui et al., 2007)	—	—	—
Amygdala (lateral)	—	178,890 (Murakami et al., 2018)	1,105 PV, 3,282 SST, 1,598 VIP (Kim et al., 2017)	—	—	—	—
Amygdala (CeA)	—	109,470 (Murakami et al., 2018)	110 PV, 23,827 SST, 145 VIP (Kim et al., 2017)	—	—	—	—
Striatum	90,158 (Baker et al., 1980) 130,000 (Steen, 2006) 73,100 (Rosen and Williams, 2001) 135,500 (Slow et al., 2003) 128,000 (Förster, 2008) 106,000 Dorsal caudate/putamen (Schmid et al., 2013) 178,000 Medial caudate/ putamen (Schmid et al., 2013) 114,700 (Andsberg et al., 2001) **Striatum Mean and STD: 120,110** ± **31,800** 108,000 D1 MSN 95,000 D2 MSN 3800 D1/D2 (Gagnon et al., 2017)	220,000 (San Jose et al., 2001) 292,000 (Förster, 2008) 127,080 (Murakami et al., 2018)	5% of total neurons, or 6,000 (Graveland and DiFiglia, 1985; Yager et al., 2015). 1,688 NADPH diaphorase (Andsberg et al., 2001) 1348 ChAT+ (Andsberg et al., 2001) 920 ChAT+ (Ransome and Turnley, 2005) 120 CR+ (Ransome and Turnley, 2005) 69 CB+ (Ransome and Turnley, 2005) 1060 PV+ (Ransome and Turnley, 2005) 1,430 PV+ (Förster, 2008) 371 PV+, 5,867 SST, 64 VIP (Kim et al., 2017) 1140 SST+ (Ransome and Turnley, 2005)	—	4,002 GFAP+ (San Jose et al., 2001) 9,000 S-100 (Steen, 2006) 4,400 (Globus pallidus) GFAP and S-100β (Charron et al., 2014) 12,000 Dorsal caudate/putamen S-100β (Schmid et al., 2013) 19,000 Medial caudate/ putamen S-100β (Schmid et al., 2013) 10,800 S-100 (Förster, 2008) **Mean and STD: 9,867** ± **5,547**	12,000(Steen, 2006) 5,300 (Förster, 2008) 12,550 (Binder et al., 2011) **Mean and STD: 9,950** ± **4,036**	12,403(San Jose et al., 2001) 15,000(Steen, 2006) 9,700 (Lawson et al., 1990) 12,100 globus pallidus (Lawson et al., 1990) 11,300 (Förster, 2008) **Mean and STD: 12,100** ± **1,900**
Nucleus Accumbens	584,000 (P1) (Bayer et al., 1994) 180,000 D1 in shell 45,000 D1/D2 in shell 298,600 all MSNs in shell 267,900 all core MSNs 45,000 D1/2 in shell (Gagnon et al., 2017)	148,280 (Murakami et al., 2018)	120 PV, 8,618 SST, 50 VIP (Kim et al., 2017)	—	–	—	1480 (Yang et al., 2013)
Thalamus	LGN 111,350 (Heumann and Rabinowicz, 1980) dLGN 152,000 (Jeffrey et al., 1995) dLGN 175,000 (Kuronen et al., 2012) dLGN 135,000 (Dursun et al., 2011) dLGN 130,000 (Scott et al., 1994) **LGN Mean and STD: 141,000** ± **24,000** dLGN 45,000 large projection neurons (Weimer et al., 2006) nucleus reticularis (RT)49,680 ± 1,097 posterior medial nucleus (POm) 41,477 ± 3,612 (Meyer et al., 2013) VPM 88,700 (Sargeant et al., 2011) VPM 77,500 (Kuronen et al., 2012) **VPM Mean and STD: 83,100** ± **7,900** VPM barreloids 51,507 ± 4,422 (Meyer et al., 2013)	122,450 (Murakami et al., 2018)	2,072 PV, 3,889 SST, 3 VIP (Kim et al., 2017)	80,966 (Heumann and Rabinowicz, 1980)	6,000 GFAP+ (Xu et al., 2007)	—	–
Hypothalamus	315,810 (Namavar et al., 2012) 320,512 (Mouton, 2014) **Mean and STD: 318,161** ± **3,325**	126,240 (Murakami et al., 2018)	905 PV, 6,768 SST, 125 VIP (Kim et al., 2017)	—	–	—	11,600 ventromedial (Lawson et al., 1990) 1,040 lateral, 910 dorsal (Yang et al., 2013)
Superior colliculus	–	—	5,329 PV+ (Pitts et al., 2013) 5,049 PV, 15,347 SST, 296 VIP (Kim et al., 2017)	—	—	—	7,200 (Lawson et al., 1990) 880 (Yang et al., 2013)
Suprachiasmatic nucleus	—	301,770 (Murakami et al., 2018)	379 PV, 5,941 SST, 13,370 VIP (Kim et al., 2017)	—	14,000 GFAP+ (Deng et al., 2010)	—	8,700 (Deng et al., 2010)
Substantia Nigra	65,451 (Timmer et al., 2007)	—	4,458 PV, 676 SST, 0 VIP (Kim et al., 2017)	—	—		13,400 (Lawson et al., 1990) 2120 (Yang et al., 2013)
Substantia Niagra pars compacta (SNpc)	20,435 TH+ (Zhang et al., 2012) 16,087 TH+ (Zhang et al., 2007) 12,600 TH+ (Bing et al., 1994) 19,700 TH+ (Bayer et al., 1994) **Mean and STD: 17,200** ± **3,600**	101,000 (Murakami et al., 2018)	4,348 CB+ (Zhang et al., 2007) 1,592 PV, 1,666 SST, 1 VIP (Kim et al., 2017)	—	—	—	—
Ventral Tegmental Area (VTA)	14,583 TH+ (Zhang et al., 2007) 14500 TH+ (Bayer et al., 1994) **Mean and STD: 14,500** ± **100**	118,100 (Murakami et al., 2018)	9,167 CB+ (Zhang et al., 2007) 3,882 PV, 1,073 SST (Kim et al., 2017)	—	—	—	880 (Yang et al., 2013)
Locus ceruleus	31,837 (Burguet et al., 2009) 19,760 (Maurin et al., 1985) **Mean and STD: 25,800** ± **8,540**	167,110 (Murakami et al., 2018)	3,777 PV, 9,223 SST, 137 VIP (Kim et al., 2017)	—	—	—	—
Subthalamic nucleus	164,479 (Sturrock, 1991)	192,460 (Murakami et al., 2018)	1,922 PV, 3,355 SST, 10 VIP (Kim et al., 2017)	—	—	—	—
Cochlear nucleus	135,824 Poly 66,321 Oct 95,759 Mul 168,483 Glob 210,282 SSPH 257,035 LSPH (Trune, 1982)	185,270 (Murakami et al., 2018)	16,349 PV, 5,584 SST, 8 VIP (Kim et al., 2017)	—	—	—	—
Spinal cord	—	—	PV, SST, VIP for all regions in (Kim et al., 2017)	7,500 (Anderson et al., 1998)	20,000 S-100β (Ohgomori et al., 2017)	3,750 (Anderson et al., 1998) 2,417 (Huang et al., 2012) **Mean and STD: 3,084** ± **943**	3,500 Iba1+ (Ohgomori et al., 2017)
Corpus callosum	—	—	—	—	—	282,398 (Duque et al., 2012) 22,319 P28, (Relucio, 2011) **Mean and STD: 152,360** ± **183,900**	—
Optic Nerve	—	—	—	—	—	197,200 (Pernet et al., 2008)	—

Olfactory bulb abbreviations: ONL, Olfactory nerve layer; GL, Glomerular layer; EPL, External plexiform layer; MCL, Mitral cell layer; IPL, Internal plexiform layer; GCL, Granule cell layer; SEL, Subependymal layer. Cell abbreviations: Poly, Polymorphic layer cell; Oct, Octopus cell; Mul, Multipolar cell; Glob, Globular cell; SSPH, Small spherical cell; LSPH, Large spherical cell. Molecular abbreviations: ChAT, Choline Acetyltransferase; TH, Tyrosine Hydroxylase.

* *The cell densities derived from (Murakami et al., 2018) are systematically lower than densities from other sources and so were not averaged in with the other densities*.

The amygdala is composed of multiple nuclei, for which estimates of neuron, interneuron, and total glia density were generally available. We were not able to find estimates for astrocyte, oligodendrocytes, and microglia for most amygdalar nuclei. However, the densities of neurons and glia for these structures have been measured in the rat (Chareyron et al., 2011). In the basolateral amygdala, the density of PV+ neurons from Fasulo agrees well with those reported by Kim, who reports PV+, SST, and VIP interneurons for all regions of the brain (Kim et al., 2017).

Striatal neurons are composed of 95% Medium Spiny Neurons, with the remaining 5% being interneurons (Graveland and DiFiglia, 1985; Yager et al., 2015). We therefore estimated interneuron density by taking 5% of the total average density of striatal neurons from Table 5, which works out to be 6,000 cells/mm^3^. This estimate of interneuron density is consistent with the sum of the interneuron subtypes found by other means (Kim et al., 2017). In the striatum the density of PV+ neurons from Forster is greater than the density reported by Kim.

The inferior colliculus can be divided into several areas, each with a particular interneuron composition (Beebe et al., 2016). However, this structure is not included in the following table since we could not identify neuron density information for each region.

Overall, subcortical structure densities are the most poorly characterized regions of the entire mouse brain. Not all subcortical structures have been sufficiently well-studied to justify their inclusion in the table. For example, we were not able to obtain good data for the septum, habenular nuclei, and pineal gland, among other structures.

### Sparsity of data

Principal cell coverage differed by region. In the hippocampus, 68% of the four cell categories searched for could be found in the sub-regions considered. In the cerebellum, 58% were found. In the cortex, 69% of seven cell categories were found, and in subcortical structures, 49% of seven cell categories were obtained. Furthermore, the amount of information per cell category differed by region (Table 6).

**Table 6:** Percent of subregions in each region for which cell category information was found.

**Region**	**Neurons**	**Non-Neuronal**	**Total cell**	**Interneurons**	**Astrocytes**	**Oligodendrocytes**	**Microglia**
Cortical	100	74	89	100	32	37	53
Hippocampal	64	—	—	50	79	—	79
Cerebellar	83	—	—	—	33	33	83
Subcortical	62	—	71	100	24	19	48

*Neurons were found in all cortical subregions studied, but astrocyte information was found in only 32 percent of cortical subregions. Oligodendrocyte density information was sparsest in subcortical structures, as only 19% has oligodendrocyte information available*.

**Figure 2:**
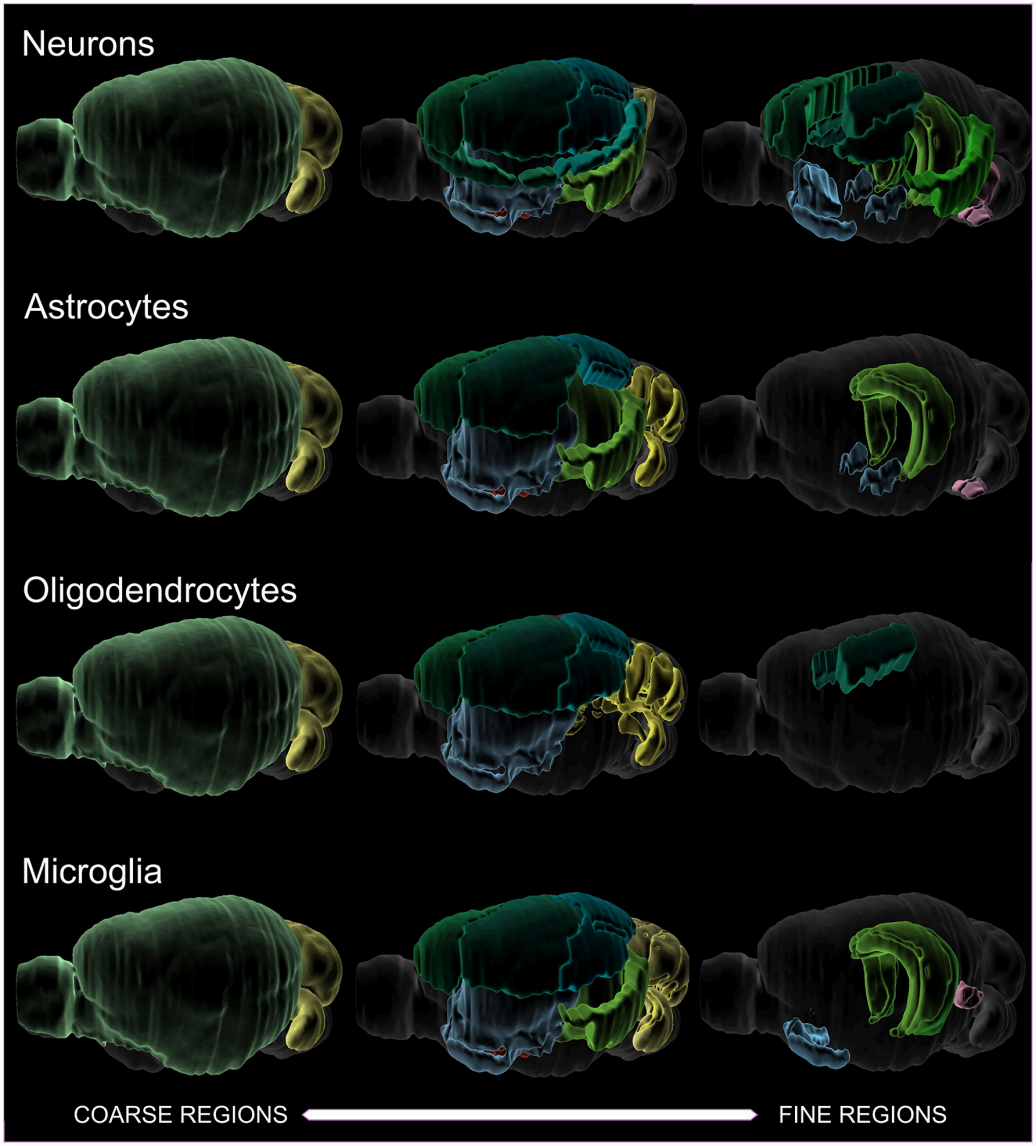
Visualization of literature densities as a function of region size, for neurons, astrocytes, oligodendrocytes, and microglia. An example of a coarse region would be cortex, while a finer region would be somatosensory barrel cortex. Oligodendrocytes have the sparsest data when finer regions are investigated. Note that total cells and various interneuron subtypes have been assayed in their entirety across the whole brain, and so are not included in this depiction. The colors serve to distinguish the boundaries of the identified regions.

When the most common cell types are plotted against the size of the region investigated (Figure 2), it is apparent that coarsely-defined regions have more data. As the target region becomes smaller, less data is available. In particular, oligodendrocyte densities are not well-characterized for finer structures. Oligodendrocyte density is, of course, highest in the white matter—as high as 85,000 cells/mm^3^ (Scafidi et al., 2014). Ultimately, the most coherent and complete data sets are obtained from scanning an entire brain (Murakami et al., 2018), since then considerations of region size are less important.

## Discussion

This review draws from the results of over100 published, experimental studies to assemble an overview of what is known about brain cell density. We found that estimates derived from different studies varied greatly, and that the current state of knowledge is still fragmented.

Experimental methods have potential sources of error. Stereology is the most rigorous approach but is also subject to observer bias in which subjective differences in cell counting arise (West, 1999). When labeled cells are used, some cell types may be undercounted when a marker expressed in only a subset of the cell population is used as a proxy for quantifying the entire population. Indeed, when multiple reports are available, a wide range of estimates is sometimes observed. While assembling the data, we found that extra transformations were sometimes needed to convert to density units, a process with the potential to introduce additional error. For example, we found that extrapolations to volumetric density from a 2D count generally were lower than densities obtained using other methods. In such extrapolations, the degree of tissue shrinkage incurred during fixation is usually not known as well, which introduces an additional source of error. Estimates of sample volume can pose a significant challenge to obtaining reliable density numbers (Oorschot, 1994).

Standard deviations of more than 50% of the mean were common when comparing the densities for the same cell type and region derived from multiple sources. In cortex, the standard deviation between neuronal density in different regions, 31,000 cells/mm^3^, is less than the average internal standard deviation within a region, 43,800 neurons/mm^3^. Therefore, the variability in different estimates of the same region is more than the variability arising from inherent differences between regions. Experimental estimates were improved in subcortical structures, as the standard deviation of individual region estimates was 19,000 neurons/mm^3^ compared to an inter-region standard deviation of 150,000 neurons/mm^3^. Subcortical structures are diverse, so much inter-regional variability is to be expected. In the hippocampus, the intra-region estimates for the same region had an average standard deviation of 96,000 neurons/mm^3^, while the inter-region standard deviation was 150,000 neurons/mm^3^. So regional estimates are still relatively useful. In the cerebellum a large variability in estimates was observed in estimates of granule cell numbers, and improved measurements would be useful in this region.

Densities for neurons and common cell types were available for the more-studied regions of the brain. However, astrocyte and oligodendrocyte density in particular were not well-characterized outside of the main brain regions. The sparseness of the data from the literature illustrates that alternative approaches are needed to fill in the gaps. One possible strategy is to use data for cell densities from related regions to fill in the gaps, or for regions at lower levels in a structural hierarchy to inherit their properties from parent regions.

Glia amounts differed by region. The ratio of astrocytes to neurons varied across the brain. It was higher in the hippocampus than in the cortex, which in turn was higher than in the cerebellum. The difference in this ratio could reflect variations in the volumetric sizes of neurons, as well as different metabolic needs. Microglia estimates typically fell in the range of 3,000–9,000 cells/mm^3^, even in regions such as the cerebellum where neuron density is high. However, some regions such as the striatum had more microglia (12,000 cells/mm^3^). Microglia are quite diverse (Grabert et al., 2016), which may be linked to the observed differences in density.

Inhibitory interneuron proportion is comparable between cortex and hippocampus. In the somatosensory cortex, the interneuron proportion is 12.3%. while in motor cortex it is 13.5% Overall in the hippocampus, the interneuron proportion is 12.5%. Although measurements for PV, SST, and VIP expressing interneurons were available for most regions, direct measurements of inhibitory interneurons were only available for limited regions. The fact that interneuron proportion does not vary markedly may help place the circuit in the proper operating regime, as inhibitory interneurons serve to counterbalance excitatory neuron activity.

Even within a region, cell densities can vary across the structure. The mapping of region names to structures used in this review does not have sufficient granularity to resolve this issue. For example, even within a named cortical layer, cell densities can vary. The most accurate density map of the brain would, therefore, not necessarily associate densities with the names of structures, but would rather measure densities at all points in a three-dimensional reference atlas. This would mitigate inhomogeneity and substructure effects and reduce subjectivity in defining brain regions.

Advances in microscopy and data processing have the potential to collect cell density information for the entire brain. Recently a quantitative brain-wide (qBrain) cell counting approach based on automated imaging by serial two-photon tomography and data analysis was developed (Kim et al., 2017). This method and a similar one (Silvestri et al., 2018) were able to produce maps of parvalbumin, somatostatin, and vasoactive intestinal peptide across the whole brain. Such an approach has the possibility to fill in many of the gaps in our current knowledge of cell densities. An expansion microscopy method has been used to identify the cell bodies of cells in the brain and construct a 3D atlas, the CUBIC-Atlas (Murakami et al., 2018). A microscopic technique that directly counts cells labeled with multiple markers has potential allow cell-type specific registration in space (Frasconi et al., 2014). In all such efforts, alignment to a standard reference atlas is critical.

Single cell transcriptomics can be used to identify genetically-defined cells in space (Codeluppi et al., 2018). As more transcriptome data becomes available, it should be possible to estimate cell densities for additional sub-types, subject to the constraint that all subtypes of a particular type of neuron should add up to the already measured density of that type. For example, the sum of all inhibitory neuron subtypes should match the total inhibitory neuron count and match densities.

In order to facilitate data sharing, it would be useful for experimentalists and theorists to come together to decide upon data presentation formats, and for journals to enforce a standardized way of presenting all stereological data. Specifying error as being either the standard error of the mean or the standard deviation, as well as sample size, would facilitate meta-analysis. Additionally, stating whether cell counts from a structure represent a single hemisphere or the entire brain would also help avoid ambiguous interpretation. In some papers, data was not reported numerically but rather in graphs and so had to be digitized to obtain a usable number. We would encourage researchers producing experimental data to take volume changes into account and provide shrinkage factors. Providing data in densities rather than custom or relative units would ensure the data is reusable. However, we recognize that experiments are designed with a question in mind and that numerical density is sometimes not an easily interpretable indicator of disease states as differences can be attributed to changes in either number or volume (West, 1999). In the end, development of a custom neuro-informatics system for researchers to deposit and share their properly-curated density numbers could enable data to be reused by the broader community.

A comprehensive knowledge of brain cell density will enable additional constraints on and validations of other aspects of brain circuit structure and function. For example, soma volumetric density is closely linked to neuronal density, and predicted soma volume fractions should align with experimentally-measured fractions. Similarly, synapse density is also closely related to type-specific neuron density, and predicted synapse densities based upon neuronal densities should be consistent with experiment measurements.

In this work, we have summarized most known estimates of brain cell density. This information will assist researchers in assessing the quality of their cell density estimates and identifying expected ranges of values. Overall, we find that for cortex, hippocampus, striatum, and cerebellum, cell densities can be reasonably constrained for principal cell types, but that for most other structures in the brain information is quite sparse. Given the limitations and inconsistencies of the literature, it is not possible to build a model of all the cell types in the brain by relying on measurements from any one source. The ultimate solution will be an integrated 3D atlas with high spatial resolution that can integrate all the data.

## Author contributions

DK collected the material. CE validated the estimates. DK and HM wrote the text.

### Conflict of interest statement

The authors declare that the research was conducted in the absence of any commercial or financial relationships that could be construed as a potential conflict of interest. The reviewer L-AD and handling Editor declared their shared affiliation.
