# Supplementary material for: Cell Densities in the Mouse Brain: A Systematic Review
Source: Front Neuroanat. 2018 Oct 23;12:83. doi: 10.3389/fnana.2018.00083 (PMC6205984; doi:10.3389/fnana.2018.00083)
Supplement: Supplementary file 1 [file Data_Sheet_1.pdf]

Supplemental Material

Calculations and Notes

Cortex

In primary auditory cortex, Basta 2005 investigated the density of neurons (Basta et al., 2005). They used 15 µm thick slices, and appear to massively underestimating the number of neurons (Table S1). We therefore excluded this study since it predicted neuronal densities of less than half that found in other regions of cortex.

Layer	Neurons per mm^2	Neurons per mm^3
I	187.5	12,500
II	492.1	32,806
III	476.3	31,753
IV	475	31,666
V	468.6	31,240
VI	472.4	31,493

Table S1. Densities derived from (Basta et al., 2005)**.**

Chen 2014 used 20 µm thick sections of sensorimotor cortex (Chen et al., 2014). They found that the density of microglia is 30/(300µm^2) section, which translates into a density of 30/ (300 * 300 * 20 *10^{-9} mm^3)= 16,667 cells/mm^3. They found that the density of nNOS neurons is 8/(1390 * 1390 *20 * 10^{-9} mm^3)= 207 cells/mm^3.

Lorke 2008 used 5 µm thick sections, and found a microglia density of 121.1 microglia/mm^2 in the somatosensory cortex, which equates to 2422 cells/mm^3. They found 112.7 microglia/mm^2 in the somatomotor cortex, or 2254 microglia/mm^3. There were 115.5 microglia /mm^2, or 2,310/mm^3 in the CA1/CA3 region. In the granular layer of the cerebellum, it was 55.8 microglia /mm^2, or 1,116 microglia /mm^3.

In cortex, Geisert found 29 glia per 15950 μm^2 x 50 μm field, which corresponds to a density of 36,363 glia/mm^3 (Geisert et al., 2002).

Angenstein tabulated densities in terms of cells/mm^2 (Angenstein et al., 2008), and using slice thicknesses of 50 μm, Therefore, each mm^2 of slice had a volume of 0.05 mm^3.

**Area**	**NeuN stained Cells**	S100β **stained cells**	**Ratio NeuN/S100**
Cortex<br>LII/III	1130/0.05=22,600	144/0.05=2,880	8.04
LIV	1609/0.05=32,180	212/0.05=4,240	7.65
LV	925/0.05=18,500	151/0.05=3,020	6.35
LVI	1201/0.05=24,020	148/0.05=2,960	8.37

Table S2. Densities derived from (Angenstein et al., 2008)**.**

In motor and somatosensory cortex, Schmid found 75 and 110 PV+ neurons per mm^2 respectively (Schmid et al., 2013). They used 50 μm thick slices, which therefore yields densities of 1,500 and 2,200 PV+ cells/mm^3.

Rockel sampled neurons in a 30 um wide by 25 um thick region across all cortex layers (Rockel et al., 1980). They report 109.2 neurons in the motor cortex sampled region, and a thickness of 1 mm. This translates to 145600 neurons/ mm^3. Rockel reports 111.9 cells in the somatosensory cortex sampled area. Fenlon gives a somatosensory adult mouse cortical thickness of 1.7 mm (Fenlon et al., 2015). Grand'Maison reports somatosensory cortex thickness of 1.1 mm (Grand’maison et al., 2013). We therefore used an average thickness of 1.4. The resulting density is 110,000 neurons/mm3. Rockel reports 110.8 cells in the frontal cortex. Grand'Maison 2013 reports frontal cortex thickness of 0.76 mm, so the resulting density is 190,000 cells/mm^3. Rockel reports 110.5 cells in the temporal cortex. Grand'Maison found a thickness of 0.88 mm so the resulting density is 170,000 cells/mm^3 (Grand’maison et al., 2013). Rockel reports 104.7 cells in the parietal sampled region and a thickness of 0.6 mm, which translates to 230,000 neurons/mm^3. Rockel reports 112.2 neurons per sampling region in visual cortex. Using their thickness of 0.7 mm for mouse primary visual cortex gives 214,000 neurons/mm^3. Rockel found an 18% linear shrinkage factor for human tissue, but did not report the shrinkage factor for mouse. It can be expected that the densities uncorrected for shrinkage are higher than they would otherwise be.

This is a provisional file, not the final typeset article

Cragg found 92,400 neurons per mm3 in mouse visual cortex (Cragg, 1967).

Heumann measured primary visual cortex of the mouse at 194,000 neurons per mm^3 (Heumann et al., 1977).

Barrera found 0.0013559 oligodendrocytes/μm^2 in the barrel hollow, and 0.001608 cells/μm^2 in the septa/barrel wall (Barrera et al., 2013). They used 50 µm sections, so the densities are 27,118 and 32,160 for and total average of 29,674 cells/mm^3.

Doucette found 20 oligodendrocyte lineage cells/500 μm^2 with 8 µm thick sections (Doucette et al., 2010). The resulting density inferred was unrealistically high.

Hill measured S100β astrocytes in superficial layers of somatosensory cortex (Hill et al., 2014). The mean was 9,548 cells/mm^3 and 2,589 oligodendrocytes/mm^3.

White matter

Scafidi found 85 oligodendrocytes / (1e6 um^3), or 85,000 cells/mm^3 in the white matter (Scafidi et al., 2014).

Tremblay found the density of microglia and oligodendrocytes using an average of 65-75 nm thick sections (Tremblay et al., 2012). They found 1.0 and 0.4 oligodendrocytes/10,000 μm^2 field of view in the auditory and visual cortices respectively. We assume an effective sampling thickness corresponding to an assumed diameter of 10 µm per cell. This is because cell somas that intersect the thin section in this diameter range are assumed to be counted. Such a correction leads to densities of 10,000 and 4,000 cells/mm^3. For microglia light microscopy using 40 µm thick slices they found 300 and 290 microglia/mm^2 for auditory and visual cortices respectively, which corresponds to densities of 7,500 and 7,250 microglia/mm^3.

Optic Nerve

Pernet found 1,972 oligodendrocytes/mm^2 in the optic nerve, using 10 µm thick sections, which implies a density of 197,200 oligodendrocytes/mm^3 (Pernet et al., 2008).

Striatum

Andsberg quantifies the total number of NeuN-immunoreactive neurons as 802679 ± 31665, the parvalbumin-containing interneurons as 11,231 ± 1278, the NADPH-diaphorase-stained interneurons as 11,817 ± 1191, and the ChAT-positive interneurons as 9,437 ± 985 (Andsberg et al., 2001). These numbers are for one side of the brain only. Assuming a whole brain striatal volume of 14 mm^3 (Stearns et al., 2007) this results in a density of 114,700 cells/mm^3 for Neuron-immunoreactive neurons, 1,688 for NADPH-diaphorase-stained interneurons and 1,348 cells/mm^3 for ChAT-positive interneurons. Striatal volume estimates will vary according to source.

Baker quantified BALB/cJ and CBA/J neuronal densities in the striatum. We averaged these together to obtain a final number: (90,593+89,722)/2 neurons/mm^3=90,157.5 neurons/mm^3 (Baker et al., 1980).

Binder found a density of 1,250 and 1,260 GST+ oligodendrocytes/mm^2 in rostral and caudal striatum respectively, using a slice thickness of 10 µm. Therefore, the densities are 12,500 and 126,000 oligodendrocytes/mm^3 respectively, for a mean of 12,550 oligodendrocytes/mm^3 (Binder et al., 2011)

Pott measured cells densities in the basal ganglia using a slice thickness of 5 µm. Assuming the cells have a diameter of 10 µm, and that any soma which protrudes into the volume is counted, we can estimate the volume density (Pott et al., 2009).

Cell Type	Medial cells/mm^2	Medial Density cells/mm^3	Lateral cells/mm^2	Lateral Density cells/mm^3	Mean Density cells/mm^3
Microglia	60	4,000	80	5,333	4,666
Oligodendrocytes	210	14,000	91	6,066	10,033
Astrocytes	5.1	340	1.2	80	210

Table S3. Densities derived from (Pott et al., 2009)**.**

These values seem much different from other reported values, and they were less than half than what was found in other studies of the basal ganglia. We therefore excluded this study.

This is a provisional file, not the final typeset article

Rauskolb found 0.00148 oligodendrocytes/um^2 in the striatum, using 60-70nm thick sections (Rauskolb et al., 2010). Assuming that oligodendrocytes have a diameter of 10 µm, and that all cells that pass through the center of the counting plane are counted, this corresponds to a density of 148,000 oligodendrocytes /mm^3. This seems quite high. We therefore excluded this study since it was more than twice the density found in other studies of the striatum.

Amygdala

Morris et al. found volumes of 0.296 and 0.3296 mm^3 for the left and right posterodorsal nucleus of the medial amygdala (MePD) in males, and 0.1768 and 0.1824 mm^3 for females (Morris et al., 2008). They found neuron numbers of 29,061 and 26,612 for the left and right MePD in males, and 21,551 and 16,326 for females, which correspond to densities of 98,179 and 80,740 neurons/mm^3 for males and 121,890 and 145,900 neurons/mm^3 for females. The overall average density is therefore 111,600 neurons/mm^3. Similarly, for glia, they found absolute numbers of 15,860 and 23,704 for the left and right MePD in males, and 12,507 and 13,134 for females, which correspond to densities of 53,581 and 71,917 glia/mm^3 for males and 70,741 and 72,007 glia/mm^3 for females. The overall average glia density is 67,062 glia/mm^3.

Locus ceruleus

Burguet et al. found 1,873 neuronal cell bodies in the locus ceruleus at P30 and 1,349 at P90 (Burguet et al., 2009). If one assumes the number declines linearly with age, at P60 the number would be 1611. This corresponds to a density of 31,837 neurons/mm^3 if a volume of 0.0506 mm^3 is used (Lein et al., 2007).

This locus ceruleus results compares to (Maurin et al., 1985) who used immune-histochemistry to count 947 neurons and histofluorescence to label 1,055 neurons, corresponding to densities of 18,694 and 20,826 with an average of 19,760 neurons/mm^3.

Hippocampus

Using the results from Fabricius 2008 we calculated the density of neurons by dividing the number of neurons by the volume of the region (Fabricius et al., 2008).

**Region**	**Density (neurons/mm^3)**
Subiculum	181/2.51= 72.11
CA4	27.9/0.75=37.2

CA1	250/1.07=233.64
CA3	180/1.46=123.28
DG	674/1.39=484.89

Table S4. Densities derived from (Fabricius et al., 2008)**.**

Shimada quantified the number of GFAP+ astrocytes in the different regions of the hippocampus, using 50 µm thick sections (Shimada et al., 1992):

**Region**	**Count per mm^2**	**Density**
Str. lacunosum-moleculare *	2,872	57,440
Str. oriens	2,256	45,120
Str. moleculare	1,804	36,080
Str. radiatum	1,538	30,760
Str. lucidum	1,450	29,000
Hilus fascia dentata	1,412	28,240
Str. pyramidale (CAI, CA3)	180	3,600
Str. granulosum	91	1,820

Table S6. Densities derived from (Shimada et al., 1992)**.**

The mean density of these hippocampal regions is thus 29,008 astrocytes/mm^3.

Wu measured the astrocyte density in senescence-accelerated-prone and resistant mice. At three months, astrocyte density was similar for both cases. We took the SAMR1 as most representative of normal mice.

Jenrow measured neurons in the dentate gyrus and CA3 (Jenrow et al., 2006), finding a mean of 11,980 neurons/mm^3 for the dentage gyrus and 20,700 neurons/mm^3 for CA3.

Geisert measured the hippocampus volume of CB6F1 mice as 8.73 mm^3, and found 182,000 neurons, 42,000 GFAP-labelled astrocytes, 38,000 S-100B-labelled astrocytes, and 38,000 microglia in the same strain (Geisert et al., 2002). This implies a density of 20,848 neurons/mm^3, 4,811 GFAP astrocytes/mm^3, 4,353 S-100B astrocytes mm^3, and 4,353 microglia/mm^3.

This is a provisional file, not the final typeset article

Vinet found the expression of oligodendrocyte subclasses in hippocampus, using 50 µm thick slices (Vinet et al., 2010). The density varies across subregions in an intricate pattern.

Kempermann found that the total number of cells in the dentate gyrus is 239,000 (Kempermann et al., 1997). Dividing by the dentate gyrus volume that they report of 2.63 * 1e8 $µm^3$ * 10^{-9} $mm^3/µm^3$ gives a density of 9,087 cells/mm^3. Alternatively, they report a sample volume of 9,000 $µm^3$ and cells counted per sample volume is 8.14. Therefore, density is 8.14 cells/ (9000 * 10^{-9} mm^3) =904,444 cells/mm^3.

Jinno found that in the CA3 region, there is no significant difference in the densities of pyramidal neurons along the dorsoventral and transverse axes (dorsal, 165,200 /mm^3 versus ventral, 172400 pyramidal neurons/mm^3) (Jinno and Kosaka, 2010). We therefore, averaged the densities to obtain an average estimate of (165.2e3+172.4e3)/2=168,800 pyramidal neurons/mm^3.

Kurt found an average of 625,080 neurons/mm^3 in the dentate gyrus of 16-17 month old mice (Kurt et al., 2004). They found neuron densities of 154,740 neurons/mm^3 in CA3 and 302,926 neurons/mm^3 in CA1.

Wirenfeldt found the number of Mac-1-positive microglial cells in the unilateral dentate gyrus of the normal young adult male C57BL/6 mouse to be 12,300 (Wirenfeldt et al., 2003). Dividing by the volume of 3.1 mm^3 gives a density of 3,968 microglia/mm^3.

Pitts assessed PV interneuron density per mm^2 section (Pitts et al., 2013). They used 40 µm thick slices.

**Region**	**cells per mm^3**
SC	5,329
MS	1,678
CA1	1,480
CA23	2,171

DG	493
IC	4,836

Table S7. Densities from (Pitts et al., 2013)**.**

Schmalbach measured cell density in various regions (Schmalbach et al., 2015):

Hippocampus CA1 Pyramidal	1,314,815
Hippocampus PV interneurons	3,100
Hippocampus Reelin interneurons	8,200
Hippocampus S100 astro	12,226
Hippocampus microglia	2,143
Cingulate Neun neurons	154,839
Cingulate PV interneurons	8,150
Cingulate S100 astro	8,920
Cingulate microglia	6,843

Table S8. Densities from (Schmalbach et al., 2015)**.**

In the dentate gyrus of rate, Jahanshahi (Jahanshahi et al., 2009) counted 17.52 astrocytes in a volume of 20,000 um2 by 7 um, which corresponds to a density of 125,142.

This is a provisional file, not the final typeset article

Long found 18800 MAC1+ microglia /mm^3 in mouse dentate gyrus and 22300 microglia /mm^3 in CA1 (Long et al., 1998).

Thalamus

Heumann found the total number of neurons in the lateral geniculate nucleus to be 16936 at 60 days of age (Heumann and Rabinowicz, 1980). The volume was 0.1521 mm^3. Therefore, the density is 111348 neurons/mm^3. They also found that total glia number is 12315, yielding a total density is 80966 glia/mm^3.

Yamada counted the number of neurons in a sampling volume (Yamada et al., 2001). For a volume of 50x50x50 um in the VPL, 63.4 cells were found, which translates to a density of 507,000 neurons/ mm^3. For a volume of 50x50x40 um in the VPL, 60.3 cells were found, which translates to a density of 603,000 neurons/mm3. For a volume of 50x50x40 um in the ventrolateral nucleus, 38.9 cells were found, which translates to a density of 389,000 neurons/mm^3.

For 10 um thick sections of the VPM, Sargeant (Sargeant et al., 2011) found 886.9 cells/mm2, which translates to a density of 88,700 cells/mm^3.

Kuronen (Kuronen et al., 2012) found 34150 neurons at 1 months and 34,830 at 3 months, which extrapolates to 34,500 at 2 months. Assuming a VPM volume of 0.445359375 mm3, this translates to a density of 77,500 cells/mm^3. In the dorsal LGN 23,600 neurons were present at 1 month and 20,300 at 5 mo, which extrapolated to 2 months is 23,000 cells. Assuming a dorsal LGN volume of 0.1314 mm^3, this translates to a density of 175,000 cells/mm3.

Jeffrey found 20,000 neurons total in dLGN (Jeffrey et al., 1995). Assuming a volume of 0.1314 mm^3, this corresponds to a density of 152,000 neurons/mm^3.

Scott found 17,000 neurons total in dLGN (Scott et al., 1994). Assuming a volume of 0.1314 mm^3, this corresponds to a density of 130,000 neurons/mm^3.

Dursun found 17700 neurons total in dLGN at P20 (Dursun et al. 2011) . Assuming a volume of 0.1314 mm^3, this corresponds to a density of 135,000 neurons/mm^3.

In the dLGN, Weimer found 3750 large projection neurons in a volume of 8.2e7 um3 in 129/SvJ mice (Weimer et al. 2006). This corresponds to a density of 45,000 cells/mm^3.

Lateral Vestibular Nucleus

Sturrock counted 744 neurons in the lateral vestibular nucleus (Sturrock, 1989). Dividing by the volume of 0.5/2=0.25 mm^3 (Lein et al. 2007) for a single nucleus results in a density of 2,976 neurons/mm^3.

Subthalamic nucleus

Sturrock counted 5,288 neurons in the subthalamic at six months of age (Sturrock, 1991). Dividing by the single nucleus volume of 0.0643/2 mm^3 (Lein et al. 2007) gives a density of 164,479 cells/mm^3.

Substantia nigra and VTA

Zhang found 3,700 tyrosine hydroxylase positive neurons (interpreted to be dopaminergic neurons) in SNpc at P28 and 3,500 in the VTA (Zhang et al., 2007). Dividing by the volumes of 0.230 mm^3 and 0.240 mm^3 yields a density of 16,087 and 14,583 dopaminergic neurons/mm^3. The number of calbindin positive cells was 1,000 and 2,200 in the sNpc and VTA, corresponding to densities of 4,348 and 9,167 cells/mm^3.

Zhang found 4,700 tyrosine hydroxylase positive neurons in the SNpc, in a volume of 0.230 mm^3, which corresponds to a density of 20,435 neurons/mm^3. (Zhang et al., 2012).

Bing found 2,900 TH positive neurons in SNpc. Dividing by the volumes of 0.230 mm^3 gives a density of 12,600 neurons/mm^3(Bing et al., 1994).

Bayer found 4,520 TH positive neurons at P1 in SNpc and 3,472 in the VTA (Bayer et al., 1994). Dividing by the volumes of 0.230 mm^3 and 0.240 mm^3 from Zhang (Zhang et al., 2007) yields a density of 19,700 neurons/mm^3 in the sNpc and 14,500 neurons/mm^3 in the VTA.

Spinal Cord

Anderson found 60 glia/mm^2 and 30 oligodendrocytes/mm^2 in spinal cord (Anderson et al., 1998). Each slice is 8 μm thick. Therefore, the density is 7,500 and 3,750 cells/mm^3, respectively.

Huang found 29 oligodendrocytes/mm^2 using 12 μm sections (Huang et al., 2012), which corresponds to a density of 2,417 oligodendrocytes/mm^3.

This is a provisional file, not the final typeset article

Olfactory Bulb

Caggiano used 30 μm sections of rat olfactory bulb to find microglia density per 1,000 μm^2 (Caggiano and Brunjes, 1993).

Region	Ramified	Ramified Density	Ameboid	Ameboid Density
SBE	0.219	7,300	0.158	5,266
GCL	0.20	6,666	0.015	500
MCL	0.23	7,666	--	--
EPL	0.28	9,333	0.015	500
GLM	0.2	6,666	--	--

Table S9. Densities from (Caggiano and Brunjes, 1993)**, in microglia/mm^3.**

Richard used 20 μm sections of olfactory bulb. In the glomerular layer, densities of NeuN+ and TH+ PG cells at two months were 1,077 cells/mm^2 for NeuN and PG cells, and 318 cells/mm^2 for TH+ PG cells, with corresponding densities of 53,850 and 15,900 cells/mm^3. (Richard et al., 2010).

Corpus callosum

Reyes-Haro measured rat corpus callosum astrocyte and glia densities at P30 (Reyes-Haro et al., 2013). They used 30 μm thick sections. Accounting for section thickness and averaging the Genu, Body, and Splenum together leads to an average astrocytes density of 4,810 and total glia density of 55,700 cells/mm^3.

Cochlear Nucleus

Trune measured neuron density in the cochlear nucleus, using 10 μm sections (Trune, 1982).

Neuron Type	Neurons/10000 μm^2	Neurons/mm^3
Poly	13.58	135,824

Oct	6.63	66,321
Mul	9.58	95,759
Glob	16.85	168,483
SSPH	21.03	210,282
LSPH	25.70	257,035

Table S10. Densities from (Trune, 1982)**.**

Cerebellum

Vela measured the microglia density in several regions of the cerebellum (Vela et al., 1995).

**Region**	**Microglia/mm^3**
ML	1,367
GL	3,281
WM	3,992
VN	5,632

Table S11. Densities from (Vela et al., 1995)**.**

Zanjani found the granule cell density to be 105 cells per volume of 16,000 μm^3, or 6,562,500 cells/mm^3 (Zanjani et al., 1997). They found the number of inferior olivary neurons to be 14,250, which assuming a volume of 0.58 mm^3 for the inferior olivary complex (Lein et al 2007) results in a density of 24,598 neurons/mm^3. Zanjani found 25e6 granule cells per hemisphere, and a granule cell/ purkinje cell ratio of 235, which implies a PC number of 213,000. Since the Purkinje cells are found in a 2D sheet, it is more appropriate to describe them in number/area units rather than number/volume. We used the Allen Institute Brain region annotation dataset to estimate the area of the purkinje cell containing layer to be 176.2 mm^2 for the whole brain. Therefore, the density of PCs is 1210 cells/ mm^2.

Caddy found that the mean number of Purkinje cells between 10 and 730 days was 177,000, and using purkinje cell containing layer to be 176.2 mm^2 the PC density is 1000 cells/mm2. At P26 the granule cell number was 27 million and at 730 days 28 million (Caddy and Biscoe, 1979). The mean

This is a provisional file, not the final typeset article

number of olive neurons between 14 and 730 days post-natal was 32,700. They found that the ratio of Purkinje cells to olive cells is ca. 5.4:1, of granule cells to Purkinje cells is ca. 170:1, of Purkinje cells to deep cerebellar nuclei neurons is ca. 10:1, and of olive neurons to deep cerebellar nuclei neurons is ca. 1.85:1.

In C57 BL/6 at age 3-4 months, Hadj-Sahraoui found 149,000 Purkinje neurons (Hadj-Sahraoui et al., 1996), which leads to a density of 846 cells/mm^2 when taking the purkinje cell containing layer to be 176.2 mm^2.

Fan found 222,600 PCs (Fan et al., 2001), which leads to a density of 1263 cells/mm^2 when taking the purkinje cell containing layer to be 176.2 mm^2.

Doulazmi found 185,000 PCs (Doulazmi et al., 1999), which leads to a density of 1050 cells/mm^2 when taking the purkinje cell containing layer to be 176.2 mm^2.

Woodruff-Pak found 194,000 PCs (Woodruff-Pak, 2006), which leads to a density of 1100 cells/mm^2 when taking the purkinje cell containing layer to be 176.2 mm^2.

Isaacs measured 216,795 PCs (Isaacs and Abbott, 1995), which leads to a density of 1230 cells/mm^2 when taking the purkinje cell containing layer to be 176.2 mm^2.

Foerster measured cell densities in various regions (Förster, 2008) :

Region	Cell Type	Density in cells/mm^3
Cerebellum	CNPase+ oligodendrocytes	15,000
	Purkinje neurons	17,000
	Stellate and Basket cells	14,000
Cerebellum granular	CNPase+ oligodendrocytes	5,400
	Iba+ microglia	3,500

	S100 astrocyte	5,400
Molecular	Iba+ microglia	3,500
Striatum	Iba+ microglia	11,300
	S100 astrocyte	10,800
	CNPase+ oligodendrocyte	5,300
	PV+ cells	1,430
	All neurons	128,000
	All Cells	292,000

Table S12. Densities obtained from (Förster, 2008)**.**

Inferior Olive

Cunningham tabulated neuron and astrocyte numbers and volume in the rat inferior olive (Cunningham et al., 1999). At P30, neuron numbers were 9495, 4397, and 3876 in the medial accessory olivary nucleus (MAO), dorsal accessory olivary nucleus (DAO), primary olivary nucleus (PO) and 22260 overall. Astrocyte numbers were 7403, 3815, 3343, and 14758 in the same regions. The volumes were .1553, .0740, .0697, and .299 mm^3. The corresponding neuron densities were 61140 , 59419, 55610 , and 74448 and the astrocyte densities were 47669, 51554, 47963, and 49358 astrocytes/mm^3.

Hypothalamus

Mouton found 750,000 neurons in the hypothalamus (Mouton, 2014). Dividing by the volume of (1.18+1.16)/2=2.34 mm^3 (Namavar et al., 2012) gives a density of 320,512 neurons/mm^3.

This is a provisional file, not the final typeset article

Validating Densities

We sought to use alternative calculations to assess the expected ranges of cell densities. Alternative estimates of astrocyte and endothelial cell density show that numbers from the literature are largely consistent.

Endothelial Cells

A geometrical approach can be used to estimate endothelial density. Brain vasculature is approximately 1.3 % of neuropil (Chklovskii et al., 2002). Capillary diameters in rat are typically 4.1 μm (Huack et al 2004, Michalaudi 2006) and in mouse they range from 3.5 to 4.0 μm (Tsai et al., 2009). For the following analysis we will therefore use a capillary thickness of 4.0 μm. The endothelial cells form the capillary and their thickness distribution extends from about 0.2 μm at regions far from their nucleus, to about 0.9 μm near the nucleus (Farkas and Luiten, 2001; Nicaise et al., 2009). If we use the lower end of the range, then the ratio of the interior area to the outer area is 91% and the percentage of capillary cross-sectional area occupied by endothelial cells is 9%. If we assume the higher end of the range, then the ratio of the interior area to the outer area is 67% and the percentage of capillaries occupied by endothelial cells becomes 33%. The microvasculature endothelial cells have an average volume of 400 μm^3 (Haas and Duling, 1997). Therefore, using this geometrical approach, the expected density should lie in the range of 2,925 endothelial cells/mm^3 to 10,725 endothelial cells/mm^3.

An alternative method of calculating density based on endothelial cell volume relies on direct measurement of the volume. The endothelial volume in the brain is only 0.2% of the total cell volume (Pardridge, 1999). Combining this with the single endothelial cell volume per cell estimate results in an estimated density of 5,000 endothelial cells/mm^3.

Pericytes

There is a 1:1 to 3:1 ratio between endothelial cells and pericytes (Mathiisen et al., 2010; Sims, 1986) so a density of 5,000 endothelial cells/mm^3 would correspond to a pericyte density between 1666 and 5,000 pericytes/mm^3.). In experiment though, there are 340 pericytes/mm^3 (Sumner 1982).

Glia

We can set an upper bound on glia density. Approximately 8-10% of neocortex neuropil volume is glia, and in the hippocampus it is 5% (Chklovskii et al., 2002; Korogod et al., 2015). S100β cells in cortex have an estimated cell body volume of 159.5 μm^3 (Garcia et al., 2010). If the cellular processes are included, the volume of a cortical astrocyte is 14700 μm^3 (Chvátal et al., 2007). This estimate might be an underestimate of the volume as confocal microscopy cannot resolve the finer details of astrocytic processes. Using the 8% volume figure with the total cell volume gives a density estimate of 5,442 astrocytes/mm^3. In practice, glia includes both oligodendrocytes and astrocytes and other glia types which could have different cell volumes.

The minimal densities for astrocytes were also computed by using counts of the projected domain volume occupied by a single astrocyte. In the cortex, astrocytes have been reported with average volumes of 21033, 36713 and 72664 μm^3 (Halassa et al., 2007) and 31000 μm^3 (Grosche et al., 2013). A domain volume of 31,000 μm^3 corresponds to a density of 32,258 astrocytes/mm^3 in cortex. In mouse hippocampus, domain volume values of 42,000 μm^3 (Grosche et al., 2013) and 85300 μm^3 have been observed (Ogata and Kosaka, 2002). In rat hippocampus a volume of 65,900 μm^3 (Bushong et al., 2002) was reported. A domain volume of 65,900 μm^3 equates to a density of 15,175/mm^3 in hippocampus. This compares well to the 16,737 astrocytes/mm^3 average obtained from the literature. These estimates should be regarded as a minimal density since although astrocytes for the most part have their own distinct spatial domains, some overlap exists between adjacent astrocytes (Ogata and Kosaka, 2002) .

Measurements of axon density in axons per surface can be related to the oligodendrocyte density in different brain regions. For example, Barrera et al. 2013 measured 0.0290 axons/um^2 in the barrel wall and 0.033 axons/um^2 in the barrel septa and corresponding implied oligodendrocyte densities of 27,118 cells/mm^3 and 32,160 cells/mm^3. This implies a cell density to axon ratio of 935,100 and 974,550, and average of 954,825 (all in units of cells/axon/mm^3 *um^2). This ratio can be applied to other areas for which the axon density is known but the oligodendrocyte density is not. The underlying assumption is that the ratio of oligodendrocytes to axon density is constant, although this may not hold true when the axon diameter varies or myelination coverage is different.

Estimates of the ratio of glia to neurons in cortex are usually 1:2 (Gabbott and Stewart, 1987). Therefore, assuming an average density of 100,000 neurons/mm^3, we would expect a glia density of 50,000 glia/mm^3. The glia are mostly composed of astrocytes and oligodendrocytes, and the sum of astrocytes and oligodendrocytes discussed above is consistent with the total glia density estimate.

Neurons

Dendrites occupy 29% of the total volume of neuropil (Braitenberg and Schüz 1991). In rats raised in an enriched environment, excitatory neurons have an average dendritic volume of 4540 μm^3 (Sirevaag and Greenough 1987). In practice, mouse neurons are expected to be smaller than rat neurons. Assuming all the neurons in the cortex were excitatory, which they are not, the excitatory neuron density would then be 63,877 neurons/mm^3.

Excitatory synapse density in cortex is about 720 million synapses/mm^3 (Schüz and Palm 1989). Excitatory neurons have a range of 5,000 to 13,000 incoming synapses per neuron, consistent with (Binzegger, Douglas, and Martin 2005). Therefore, the range of pyramidal cell densities is 55,385 to 144,000 neurons/mm^3.

In adulthood, the relative percentage of asymmetrical to symmetrical synapses is 85.4%/14.6% (De Felipe et al. 1997). Excitatory synapses tend to be asymmetric, while inhibitory ones are symmetric. If we take the asymmetrical synapse density to be 720 million synapses/mm^3, the symmetrical synapse density is 123 million synapses/mm^3. Assuming that these synapses all originate from interneurons, and that the number of outgoing synapses per interneuron is 3,000 (consistent with (Binzegger et al., 2004)) the expected density is 41,000 inhibitory neurons/mm^3.

This is a provisional file, not the final typeset article

**Type of Cell**	**Methodology**	**Estimate (cells/mm^3)**
Excitatory Neuron	Synapse	55,385 to 144,000
	Volume	63,877
Inhibitory Neuron	Synapse	41,000
Glia	Ratio to Neurons	50,000
Astrocyte	Domain Volume	32,258
	Cell Volume	5,442
Endothelial	Geometrical	2,925 to 10,725
Pericyte	Capillary Branch Points	4,596

Table S13. Summary of cortical cell density ranges obtained using different methodologies.

Total Cells

To process the data from Murakami 2018, we used the Allen Brain Atlas region hierarchy to sum the cell number of each sub-level region that constitutes each higher level region. Then we used the meshes from the Allen Brain Atlas to compute the volume of each region. The density was the number of cells divided by the volume of the region.

Comparison of Mouse and Rat

A systematic comparison of mouse and rat densities was beyond the scope of the review, but we conducted a limited survey for two areas in which more literature data is available, the somatosensory cortex and striatum.

Region	Density	Source
Somatosensory Cortex	110,200 cells/mm^3	(Ren et al., 1992)

	119,000 cells/mm^3	(Nurnberger and Gordon, 1957)
	112,000 cells/mg (116000 cells/mm3)	(Brizzee et al., 1964)
**Mean and STD**	**114,000±4,600**	
Dorsal Striatum	110,000 cells/mm^3	(Meitzen et al., 2011)
	140,150 cells/mm^3	(Oorschot, 1996)
**Mean and STD**	**125,000±20,000**	

Table S14. Rat neuron densities for somatosensory cortex and dorsal striatum.

We assumed that the specific gravity of cortex is 1.04 (DiResta et al., 1990) so that the density for the Brizzee somatosensory cortex was 116,000 cells/mm^3.

In the mouse somatosensory cortex (Table 1), we compute a mean and STD of 110,660 ± 933 versus 114,000 ± 4,600 in the above table for rat. In the mouse striatum (Table 5), we compute a mean and STD of 120,110 ± 31,800 versus 125,000 ± 20,000 in the above table for rat. The densities in these regions are not statistically different between the two species (ttest; p=0.44, 0.8219 respectively).

References

Anderson, T. J., Schneider, A., Barrie, J. A., Klugmann, M., McCulloch, M. C., Kirkham, D., et al. (1998). Late-onset neurodegeneration in mice with increased dosage of the proteolipid protein gene. *J. Comp. Neurol.* 394, 506–519.

Andsberg, G., Kokaia, Z., and Lindvall, O. (2001). Upregulation of p75 neurotrophin receptor after stroke in mice does not contribute to differential vulnerability of striatal neurons. *Exp. Neurol.* 169, 351–363. doi:10.1006/exnr.2001.7646.

Angenstein, F., Hilfert, L., Zuschratter, W., Altrock, W. D., Niessen, H. G., and Gundelfinger, E. D. (2008). Morphological and metabolic changes in the cortex of mice lacking the functional presynaptic active zone protein bassoon: a combined 1H-NMR spectroscopy and histochemical study. *Cereb. Cortex N. Y. N 1991* 18, 890–897. doi:10.1093/cercor/bhm122.

Baker, H., Joh, T. H., and Reis, D. J. (1980). Genetic control of number of midbrain dopaminergic neurons in inbred strains of mice: relationship to size and neuronal density of the striatum. *Proc. Natl. Acad. Sci. U. S. A.* 77, 4369–4373.

This is a provisional file, not the final typeset article

Barrera, K., Chu, P., Abramowitz, J., Steger, R., Ramos, R. L., and Brumberg, J. C. (2013). Organization of myelin in the mouse somatosensory barrel cortex and the effects of sensory deprivation. *Dev. Neurobiol.* 73, 297–314. doi:10.1002/dneu.22060.

Basta, D., Tzschentke, B., and Ernst, A. (2005). Noise-induced cell death in the mouse medial geniculate body and primary auditory cortex. *Neurosci. Lett.* 381, 199–204. doi:10.1016/j.neulet.2005.02.034.

Bayer, S. A., Triarhou, L. C., Thomas, J. D., and Ghetti, B. (1994). Correlated quantitative studies of the neostriatum, nucleus accumbens, substantia nigra, and ventral tegmental area in normal and weaver mutant mice. *J. Neurosci.* 14, 6901–6910. doi:10.1523/JNEUROSCI.14-11-06901.1994.

Binder, M. D., Xiao, J., Kemper, D., Ma, G. Z. M., Murray, S. S., and Kilpatrick, T. J. (2011). Gas6 increases myelination by oligodendrocytes and its deficiency delays recovery following cuprizone-induced demyelination. *PloS One* 6, e17727. doi:10.1371/journal.pone.0017727.

Bing, G., Zhang, Y., Watanabe, Y., McEwen, B. S., and Stone, E. A. (1994). Locus coeruleus lesions potentiate neurotoxic effects of MPTP in dopaminergic neurons of the substantia nigra. *Brain Res.* 668, 261–265.

Binzegger, T., Douglas, R. J., and Martin, K. A. C. (2004). A quantitative map of the circuit of cat primary visual cortex. *J. Neurosci. Off. J. Soc. Neurosci.* 24, 8441–8453. doi:10.1523/JNEUROSCI.1400-04.2004.

Brizzee, K. R., Vogt, J., and Kharetchko, X. (1964). “Postnatal Changes in Glia/Neuron Index with a Comparison of Methods of Cell Enumeration in the White Rat,” in *Progress in Brain Research* Growth and Maturation of the Brain., eds. D. P. Purpura and J. P. Schadé (Elsevier), 136–149. doi:10.1016/S0079-6123(08)61274-1.

Burguet, J., Andrey, P., Rampin, O., and Maurin, Y. (2009). Three-dimensional statistical modeling of neuronal populations: illustration with spatial localization of supernumerary neurons in the locus coeruleus of quaking mutant mice. *J. Comp. Neurol.* 513, 483–495. doi:10.1002/cne.21954.

Bushong, E. A., Martone, M. E., Jones, Y. Z., and Ellisman, M. H. (2002). Protoplasmic astrocytes in CA1 stratum radiatum occupy separate anatomical domains. *J. Neurosci. Off. J. Soc. Neurosci.* 22, 183–192.

Caddy, K. W., and Biscoe, T. J. (1979). Structural and quantitative studies on the normal C3H and Lurcher mutant mouse. *Philos. Trans. R. Soc. Lond. B. Biol. Sci.* 287, 167–201.

Caggiano, A. O., and Brunjes, P. C. (1993). Microglia and the developing olfactory bulb. *Neuroscience* 52, 717–724.

Chen, J.-R., Wang, B.-N., Tseng, G.-F., Wang, Y.-J., Huang, Y.-S., and Wang, T.-J. (2014). Morphological changes of cortical pyramidal neurons in hepatic encephalopathy. *BMC Neurosci.* 15, 15. doi:10.1186/1471-2202-15-15.

Chklovskii, D. B., Schikorski, T., and Stevens, C. F. (2002). Wiring optimization in cortical circuits. *Neuron* 34, 341–347.

Chvátal, A., Anderová, M., Hock, M., Prajerová, I., Neprasová, H., Chvátal, V., et al. (2007). Three-dimensional confocal morphometry reveals structural changes in astrocyte morphology in situ. *J. Neurosci. Res.* 85, 260–271. doi:10.1002/jnr.21113.

Cragg, B. G. (1967). The density of synapses and neurones in the motor and visual areas of the cerebral cortex. *J. Anat.* 101, 639–654.

Cunningham, J. J., Sherrard, R. M., Bedi, K. S., Renshaw, G. M., and Bower, A. J. (1999). Changes in the numbers of neurons and astrocytes during the postnatal development of the rat inferior olive. *J. Comp. Neurol.* 406, 375–383.

DiResta, G. R., Lee, J., Lau, N., Ali, F., Galicich, J. H., and Arbit, E. (1990). Measurement of brain tissue density using pycnometry. *Acta Neurochir. Suppl. (Wien)* 51, 34–36.

Doucette, J. R., Jiao, R., and Nazarali, A. J. (2010). Age-related and cuprizone-induced changes in myelin and transcription factor gene expression and in oligodendrocyte cell densities in the rostral corpus callosum of mice. *Cell. Mol. Neurobiol.* 30, 607–629. doi:10.1007/s10571-009-9486-z.

Doulazmi, M., Frédéric, F., Lemaigre-Dubreuil, Y., Hadj-Sahraoui, N., Delhaye-Bouchaud, N., and Mariani, J. (1999). Cerebellar Purkinje cell loss during life span of the heterozygous staggerer mouse (Rora(+)/Rora(sg)) is gender-related. *J. Comp. Neurol.* 411, 267–273.

Fabricius, K., Wörtwein, G., and Pakkenberg, B. (2008). The impact of maternal separation on adult mouse behaviour and on the total neuron number in the mouse hippocampus. *Brain Struct. Funct.* 212, 403–416. doi:10.1007/s00429-007-0169-6.

Fan, H., Favero, M., and Vogel, M. W. (2001). Elimination of Bax expression in mice increases cerebellar purkinje cell numbers but not the number of granule cells. *J. Comp. Neurol.* 436, 82–91.

Farkas, E., and Luiten, P. G. (2001). Cerebral microvascular pathology in aging and Alzheimer’s disease. *Prog. Neurobiol.* 64, 575–611.

Fenlon, L. R., Liu, S., Gobius, I., Kurniawan, N. D., Murphy, S., Moldrich, R. X., et al. (2015). Formation of functional areas in the cerebral cortex is disrupted in a mouse model of autism spectrum disorder. *Neural Develop.* 10, 10. doi:10.1186/s13064-015-0033-y.

Förster, J. (2008). Quantitative morphological analysis of the neostriatum and the cerebellum of tenascin-C deficient mice.

Gabbott, P. L., and Stewart, M. G. (1987). Distribution of neurons and glia in the visual cortex (area 17) of the adult albino rat: a quantitative description. *Neuroscience* 21, 833–845.

Garcia, A. D. R., Petrova, R., Eng, L., and Joyner, A. L. (2010). Sonic hedgehog regulates discrete populations of astrocytes in the adult mouse forebrain. *J. Neurosci. Off. J. Soc. Neurosci.* 30, 13597–13608. doi:10.1523/JNEUROSCI.0830-10.2010.

This is a provisional file, not the final typeset article

Geisert, E. E., Williams, R. W., Geisert, G. R., Fan, L., Asbury, A. M., Maecker, H. T., et al. (2002). Increased brain size and glial cell number in CD81-null mice. *J. Comp. Neurol.* 453, 22–32. doi:10.1002/cne.10364.

Grand'maison, M., Zehntner, S. P., Ho, M.-K., Hébert, F., Wood, A., Carbonell, F., et al. (2013). Early cortical thickness changes predict β-amyloid deposition in a mouse model of Alzheimer's disease. *Neurobiol. Dis.* 54, 59–67. doi:10.1016/j.nbd.2013.02.005.

Grosche, A., Grosche, J., Tackenberg, M., Scheller, D., Gerstner, G., Gumprecht, A., et al. (2013). Versatile and simple approach to determine astrocyte territories in mouse neocortex and hippocampus. *PloS One* 8, e69143. doi:10.1371/journal.pone.0069143.

Haas, T. L., and Duling, B. R. (1997). Morphology favors an endothelial cell pathway for longitudinal conduction within arterioles. *Microvasc. Res.* 53, 113–120. doi:10.1006/mvre.1996.1999.

Hadj-Sahraoui, N., Frédéric, F., Delhaye-Bouchaud, N., and Mariani, J. (1996). Gender effect on Purkinje cell loss in the cerebellum of the heterozygous reeler mouse. *J. Neurogenet.* 11, 45–58.

Halassa, M. M., Fellin, T., Takano, H., Dong, J.-H., and Haydon, P. G. (2007). Synaptic islands defined by the territory of a single astrocyte. *J. Neurosci. Off. J. Soc. Neurosci.* 27, 6473–6477. doi:10.1523/JNEUROSCI.1419-07.2007.

Heumann, D., Leuba, G., and Rabinowicz, T. (1977). Postnatal development of the mouse cerebral neocortex. II. Quantitative cytoarchitectonics of visual and auditory areas. *J. Hirnforsch.* 18, 483–500.

Heumann, D., and Rabinowicz, T. (1980). Postnatal development of the dorsal lateral geniculate nucleus in the normal and enucleated albino mouse. *Exp. Brain Res.* 38, 75–85.

Hill, R. A., Patel, K. D., Goncalves, C. M., Grutzendler, J., and Nishiyama, A. (2014). Modulation of oligodendrocyte generation during a critical temporal window after NG2 cell division. *Nat. Neurosci.* 17, 1518–1527. doi:10.1038/nn.3815.

Huang, J. K., Ferrari, C. C., Monteiro de Castro, G., Lafont, D., Zhao, C., Zaratin, P., et al. (2012). Accelerated axonal loss following acute CNS demyelination in mice lacking protein tyrosine phosphatase receptor type Z. *Am. J. Pathol.* 181, 1518–1523. doi:10.1016/j.ajpath.2012.07.011.

Isaacs, K. R., and Abbott, L. C. (1995). Cerebellar volume decreases in the tottering mouse are specific to the molecular layer. *Brain Res. Bull.* 36, 309–314.

Jahanshahi, M., Golalipour, M. J., and Afshar, M. (2009). The effect of Urtica dioica extract on the number of astrocytes in the dentate gyrus of diabetic rats. *Folia Morphol.* 68, 93–97.

Jeffrey, M., Fraser, J. R., Halliday, W. G., Fowler, N., Goodsir, C. M., and Brown, D. A. (1995). Early unsuspected neuron and axon terminal loss in scrapie-infected mice revealed by morphometry and immunocytochemistry. *Neuropathol. Appl. Neurobiol.* 21, 41–49.

Jenrow, K. A., Ratkewicz, A. E., Zalinski, D. N., Roszka, K. M., Lemke, N. W., and Elisevich, K. V. (2006). Influence of ionizing radiation on the course of kindled epileptogenesis. *Brain Res.* 1094, 207–216. doi:10.1016/j.brainres.2006.03.096.

Jinno, S., and Kosaka, T. (2010). Stereological estimation of numerical densities of glutamatergic principal neurons in the mouse hippocampus. *Hippocampus* 20, 829–840. doi:10.1002/hipo.20685.

Kempermann, G., Kuhn, H. G., and Gage, F. H. (1997). Genetic influence on neurogenesis in the dentate gyrus of adult mice. *Proc. Natl. Acad. Sci. U. S. A.* 94, 10409–10414.

Korogod, N., Petersen, C. C. H., and Knott, G. W. (2015). Ultrastructural analysis of adult mouse neocortex comparing aldehyde perfusion with cryo fixation. *eLife* 4. doi:10.7554/eLife.05793.

Kuronen, M., Lehesjoki, A.-E., Jalanko, A., Cooper, J. D., and Kopra, O. (2012). Selective spatiotemporal patterns of glial activation and neuron loss in the sensory thalamocortical pathways of neuronal ceroid lipofuscinosis 8 mice. *Neurobiol. Dis.* 47, 444–457. doi:10.1016/j.nbd.2012.04.018.

Kurt, M. A., Kafa, M. I., Dierssen, M., and Davies, D. C. (2004). Deficits of neuronal density in CA1 and synaptic density in the dentate gyrus, CA3 and CA1, in a mouse model of Down syndrome. *Brain Res.* 1022, 101–109. doi:10.1016/j.brainres.2004.06.075.

Lein, E. S., Hawrylycz, M. J., Ao, N., Ayres, M., Bensinger, A., Bernard, A., et al. (2007). Genome-wide atlas of gene expression in the adult mouse brain. *Nature* 445, 168–176. doi:10.1038/nature05453.

Long, J. M., Kalehua, A. N., Muth, N. J., Calhoun, M. E., Jucker, M., Hengemihle, J. M., et al. (1998). Stereological analysis of astrocyte and microglia in aging mouse hippocampus. *Neurobiol. Aging* 19, 497–503.

Mathiisen, T. M., Lehre, K. P., Danbolt, N. C., and Ottersen, O. P. (2010). The perivascular astroglial sheath provides a complete covering of the brain microvessels: an electron microscopic 3D reconstruction. *Glia* 58, 1094–1103. doi:10.1002/glia.20990.

Maurin, Y., Berger, B., Le Saux, F., Gay, M., and Baumann, N. (1985). Increased number of locus ceruleus noradrenergic neurons in the convulsive mutant quaking mouse. *Neurosci. Lett.* 57, 313–318.

Meitzen, J., Pflepsen, K. R., Stern, C. M., Meisel, R. L., and Mermelstein, P. G. (2011). Measurements of neuron soma size and density in rat dorsal striatum, nucleus accumbens core and nucleus accumbens shell: differences between striatal region and brain hemisphere, but not sex. *Neurosci. Lett.* 487, 177–181. doi:10.1016/j.neulet.2010.10.017.

Morris, J. A., Jordan, C. L., and Breedlove, S. M. (2008). Sexual dimorphism in neuronal number of the posterodorsal medial amygdala is independent of circulating androgens and regional volume in adult rats. *J. Comp. Neurol.* 506, 851–859. doi:10.1002/cne.21536.

Mouton, P. R. ed. (2014). *Neurostereology: unbiased stereology of neural systems*. Ames, Iowa: Wiley Blackwell.

This is a provisional file, not the final typeset article

Namavar, M. R., Raminfard, S., Jahromi, Z. V., and Azari, H. (2012). Effects of high-fat diet on the numerical density and number of neuronal cells and the volume of the mouse hypothalamus: a stereological study. *Anat. Cell Biol.* 45, 178–184. doi:10.5115/acb.2012.45.3.178.

Nicaise, C., Mitrecic, D., Demetter, P., De Decker, R., Authelet, M., Boom, A., et al. (2009). Impaired blood-brain and blood-spinal cord barriers in mutant SOD1-linked ALS rat. *Brain Res.* 1301, 152–162. doi:10.1016/j.brainres.2009.09.018.

Nurnberger, J. I., and Gordon, M. W. (1957). The cell density of neural tissues: direct counting method and possible applications as a biologic referent. *Prog. Neurobiol.* 2, 100-128; discussion 128-138.

Ogata, K., and Kosaka, T. (2002). Structural and quantitative analysis of astrocytes in the mouse hippocampus. *Neuroscience* 113, 221–233.

Oorschot, D. E. (1996). Total number of neurons in the neostriatal, pallidal, subthalamic, and substantia nigral nuclei of the rat basal ganglia: a stereological study using the cavalieri and optical disector methods. *J. Comp. Neurol.* 366, 580–599. doi:10.1002/(SICI)1096-9861(19960318)366:4<580::AID-CNE3>3.0.CO;2-0.

Pardridge, W. M. (1999). Blood-brain barrier biology and methodology. *J. Neurovirol.* 5, 556–569. doi:10.3109/13550289909021285.

Pernet, V., Joly, S., Christ, F., Dimou, L., and Schwab, M. E. (2008). Nogo-A and myelin-associated glycoprotein differently regulate oligodendrocyte maturation and myelin formation. *J. Neurosci. Off. J. Soc. Neurosci.* 28, 7435–7444. doi:10.1523/JNEUROSCI.0727-08.2008.

Pitts, M. W., Reeves, M. A., Hashimoto, A. C., Ogawa, A., Kremer, P., Seale, L. A., et al. (2013). Deletion of selenoprotein M leads to obesity without cognitive deficits. *J. Biol. Chem.* 288, 26121–26134. doi:10.1074/jbc.M113.471235.

Pott, F., Gingele, S., Clarner, T., Dang, J., Baumgartner, W., Beyer, C., et al. (2009). Cuprizone effect on myelination, astrogliosis and microglia attraction in the mouse basal ganglia. *Brain Res.* 1305, 137–149. doi:10.1016/j.brainres.2009.09.084.

Rauskolb, S., Zagrebelsky, M., Dreznjak, A., Deogracias, R., Matsumoto, T., Wiese, S., et al. (2010). Global deprivation of brain-derived neurotrophic factor in the CNS reveals an area-specific requirement for dendritic growth. *J. Neurosci. Off. J. Soc. Neurosci.* 30, 1739–1749. doi:10.1523/JNEUROSCI.5100-09.2010.

Ren, J. Q., Aika, Y., Heizmann, C. W., and Kosaka, T. (1992). Quantitative analysis of neurons and glial cells in the rat somatosensory cortex, with special reference to GABAergic neurons and parvalbumin-containing neurons. *Exp. Brain Res.* 92, 1–14. doi:10.1007/BF00230378.

Reyes-Haro, D., Mora-Loyola, E., Soria-Ortiz, B., and García-Colunga, J. (2013). Regional density of glial cells in the rat corpus callosum. *Biol. Res.* 46, 27–32. doi:10.4067/S0716-97602013000100004.

Richard, M. B., Taylor, S. R., and Greer, C. A. (2010). Age-induced disruption of selective olfactory bulb synaptic circuits. *Proc. Natl. Acad. Sci. U. S. A.* 107, 15613–15618. doi:10.1073/pnas.1007931107.

Rockel, A. J., Hiorns, R. W., and Powell, T. P. (1980). The basic uniformity in structure of the neocortex. *Brain J. Neurol.* 103, 221–244.

Sargeant, T. J., Wang, S., Bradley, J., Smith, N. J. C., Raha, A. A., McNair, R., et al. (2011). Adeno-associated virus-mediated expression of β-hexosaminidase prevents neuronal loss in the Sandhoff mouse brain. *Hum. Mol. Genet.* 20, 4371–4380. doi:10.1093/hmg/ddr364.

Scafidi, J., Hammond, T. R., Scafidi, S., Ritter, J., Jablonska, B., Roncal, M., et al. (2014). Intranasal epidermal growth factor treatment rescues neonatal brain injury. *Nature* 506, 230–234. doi:10.1038/nature12880.

Schmalbach, B., Lepsveridze, E., Djogo, N., Papashvili, G., Kuang, F., Leshchyns'ka, I., et al. (2015). Age-dependent loss of parvalbumin-expressing hippocampal interneurons in mice deficient in CHL1, a mental retardation and schizophrenia susceptibility gene. *J. Neurochem.* 135, 830–844. doi:10.1111/jnc.13284.

Schmid, J. S., Bernreuther, C., Nikonenko, A. G., Ling, Z., Mies, G., Hossmann, K.-A., et al. (2013). Heterozygosity for the mutated X-chromosome-linked L1 cell adhesion molecule gene leads to increased numbers of neurons and enhanced metabolism in the forebrain of female carrier mice. *Brain Struct. Funct.* 218, 1375–1390. doi:10.1007/s00429-012-0463-9.

Scott, J. R., Jeffrey, M., and Halliday, W. G. (1994). Unsuspected early neuronal loss in scrapie-infected mice revealed by morphometric analysis. *Ann. N. Y. Acad. Sci.* 724, 338–343.

Shimada, M., Akagi, N., Goto, H., Watanabe, H., Nakanishi, M., Hirose, Y., et al. (1992). Microvessel and astroglial cell densities in the mouse hippocampus. *J. Anat.* 180 (Pt 1), 89–95.

Sims, D. E. (1986). The pericyte--a review. *Tissue Cell* 18, 153–174.

Stearns, N. A., Schaevitz, L. R., Bowling, H., Nag, N., Berger, U. V., and Berger-Sweeney, J. (2007). Behavioral and anatomical abnormalities in Mecp2 mutant mice: a model for Rett syndrome. *Neuroscience* 146, 907–921. doi:10.1016/j.neuroscience.2007.02.009.

Sturrock, R. R. (1989). Age related changes in neuron number in the mouse lateral vestibular nucleus. *J. Anat.* 166, 227–232.

Sturrock, R. R. (1991). Stability of neuron number in the subthalamic and entopeduncular nuclei of the ageing mouse brain. *J. Anat.* 179, 67–73.

Tremblay, M.-È., Zettel, M. L., Ison, J. R., Allen, P. D., and Majewska, A. K. (2012). Effects of aging and sensory loss on glial cells in mouse visual and auditory cortices. *Glia* 60, 541–558. doi:10.1002/glia.22287.

This is a provisional file, not the final typeset article

Trune, D. R. (1982). Influence of neonatal cochlear removal on the development of mouse cochlear nucleus: I. Number, size, and density of its neurons. *J. Comp. Neurol.* 209, 409–424. doi:10.1002/cne.902090410.

Tsai, P. S., Kaufhold, J. P., Blinder, P., Friedman, B., Drew, P. J., Karten, H. J., et al. (2009). Correlations of neuronal and microvascular densities in murine cortex revealed by direct counting and colocalization of nuclei and vessels. *J. Neurosci. Off. J. Soc. Neurosci.* 29, 14553–14570. doi:10.1523/JNEUROSCI.3287-09.2009.

Vela, J. M., Dalmau, I., González, B., and Castellano, B. (1995). Morphology and distribution of microglial cells in the young and adult mouse cerebellum. *J. Comp. Neurol.* 361, 602–616. doi:10.1002/cne.903610405.

Vinet, J., Lemieux, P., Tamburri, A., Tiesinga, P., Scafidi, J., Gallo, V., et al. (2010). Subclasses of oligodendrocytes populate the mouse hippocampus. *Eur. J. Neurosci.* 31, 425–438. doi:10.1111/j.1460-9568.2010.07082.x.

Wirenfeldt, M., Dalmau, I., and Finsen, B. (2003). Estimation of absolute microglial cell numbers in mouse fascia dentata using unbiased and efficient stereological cell counting principles. *Glia* 44, 129–139. doi:10.1002/glia.10277.

Woodruff-Pak, D. S. (2006). Stereological estimation of Purkinje neuron number in C57BL/6 mice and its relation to associative learning. *Neuroscience* 141, 233–243. doi:10.1016/j.neuroscience.2006.03.070.

Yamada, A., Saji, M., Ukita, Y., Shinoda, Y., Taniguchi, M., Higaki, K., et al. (2001). Progressive neuronal loss in the ventral posterior lateral and medial nuclei of thalamus in Niemann–Pick disease type C mouse brain. *Brain Dev.* 23, 288–297. doi:10.1016/S0387-7604(01)00209-1.

Zanjani, H. S., Vogel, M. W., Delhaye-Bouchaud, N., Martinou, J. C., and Mariani, J. (1997). Increased inferior olivary neuron and cerebellar granule cell numbers in transgenic mice overexpressing the human Bcl-2 gene. *J. Neurobiol.* 32, 502–516.

Zhang, J., Pho, V., Bonasera, S. J., Holtzman, J., Tang, A. T., Hellmuth, J., et al. (2007). Essential function of HIPK2 in TGFbeta-dependent survival of midbrain dopamine neurons. *Nat. Neurosci.* 10, 77–86. doi:10.1038/nn1816.

Zhang, Y., Granholm, A.-C., Huh, K., Shan, L., Diaz-Ruiz, O., Malik, N., et al. (2012). PTEN deletion enhances survival, neurite outgrowth and function of dopamine neuron grafts to MitoPark mice. *Brain J. Neurol.* 135, 2736–2749. doi:10.1093/brain/aws196.
